# Comparative proteomic analysis reveals the effects of different light spectra on protein expression in *Hericium erinaceus* mycelium

**DOI:** 10.3389/ffunb.2026.1791721

**Published:** 2026-03-09

**Authors:** Siripong Sukdee, Ornprapa Thepsilvisut, Jatuphol Pholtaisong, Onmanee Prajuabjinda, Preuk Chutimanukul

**Affiliations:** 1 Department of Biotechnology, Faculty of Science and Technology, Thammasat University, Khlong Luang, Pathumthani, Thailand; 2 Department of Agricultural Technology, Faculty of Science and Technology, Thammasat University, Khlong Luang, Pathumthani, Thailand; 3 Thammasat University Center of Excellence in Global Food Security, Thammasat University, Khlong Luang, Pathumthani, Thailand; 4 Department of Applied Thai Traditional Medicine, Faculty of Medicine, Thammasat University, Khlong Luang, Pathumthani, Thailand

**Keywords:** differentially expressed proteins (DEPs), LED light, medicinal mushroom, photoreceptor protein, proteomics

## Abstract

**Introduction:**

Light represents a major environmental factor influencing the growth, developmental programming, and metabolic regulation of *Hericium erinaceus*. Different wavelengths differentially affect mycelial development, stress responses, and protein expression, highlighting the complexity of fungal photoregulation. However, the molecular mechanisms and light-responsive regulatory networks in *H. erinaceus* remain largely unclear, limiting our understanding of how specific light cues shape its proteomic profiles.

**Methods:**

A label-free LC-MS/MS quantitative proteomics approach was employed to investigate the global protein expression profiles of *H. erinaceus* mycelia growing under different light treatments, including blue, green, red, and RGB qualities, compared to control (darkness). The differentially expressed proteins (DEPs) were subsequently annotated and analyzed using the Gene Ontology (GO) and Kyoto Encyclopedia of Genes and Genomes (KEGG) databases.

**Results:**

In this study, a total of 4,618 proteins were identified in *H. erinaceus*, of which 560 were expressed across all experimental conditions. Comparative proteomic analysis under different light treatments revealed 550–677 DEPs per condition, with the blue-light treatment exhibiting the greatest number of uniquely expressed proteins. Light exposure modulated GO-enriched metabolic, biosynthetic, and enzymatic functions in *H. erinaceus*. RGB induced the broadest responses, while blue, green, and red produced distinct wavelength-specific regulatory patterns. KEGG pathway analysis showed wavelength-dependent proteomic shifts in *H. erinaceus*, with RGB inducing the strongest metabolic and signaling responses, while blue, green, and red differentially activated energy, biosynthesis, and regulatory pathways. These results support the molecular-mechanistic approach employed and offer valuable insights into protein expression dynamics and regulatory pathways, while also clarifying how different light qualities influence the developmental processes of *H. erinaceus*.

## Graphical Abstract

**Figure:**
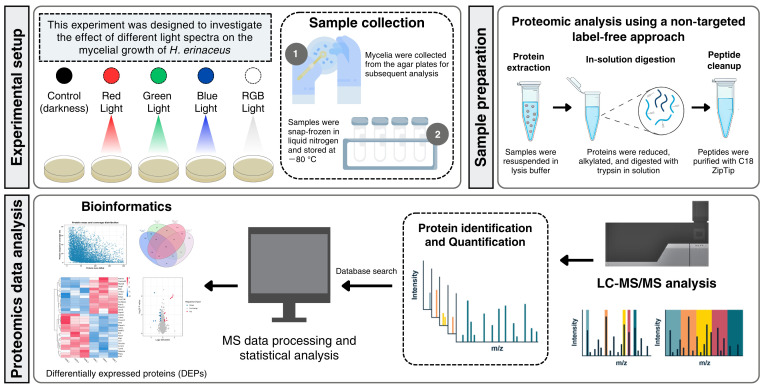

## 1 Introduction

The species *Hericium erinaceus* (Bull.:Fr) Pers., commonly known as Lion’s mane or Yamabushitake mushroom, is a medicinal and culinary fungus recognized for its neuroprotective, antioxidant, and anticancer properties (Chutimanukul et al., 2023b). These bioactivities are attributed to the presence of bioactive compounds such as hericenones and erinacines, which have shown potential in promoting nerve growth factor (NGF) synthesis and modulating neuronal functions (Li et al., 2018; Chen et al., 2024). In addition, *H. erinaceus* has attracted the attention of numerous researchers in the past few years owing to its beneficial medical properties (Wong et al., 2013; Chutimanukul et al., 2023a). The biosynthesis of these secondary metabolites is influenced by various environmental conditions, including temperature, nutrient composition, and notably, light exposure (Mykchaylova et al., 2023). Among environmental cues, light serves as a major determinant of fungal behavior, orchestrating critical processes such as developmental decisions (including fruiting body formation and sporulation), stress responses, physiological adaptations, metabolism, and circadian regulation (Nesatyy and Suter, 2007; Corrochano, 2019).

Light is a fundamental energy source that drives various biological processes across all living organisms on earth. In fungi, it plays a pivotal role in regulating essential processes such as growth, morphogenesis, directional development, reproduction, and secondary metabolite production, all of which are vital for their survival and dispersal (Yu and Fischer, 2019; Chutimanukul et al., 2025). Different wavelengths of light, including red, blue, and green, trigger specific physiological responses in fungi, mediated by specialized photoreceptors like cryptochromes, phytochromes, and white-collar proteins (Bayram et al., 2008; Corrochano, 2019). Extensive studies on filamentous fungi, such as *Neurospora crassa* and *Aspergillus nidulans*, revealed that these organisms sense and respond to diverse light intensities and wavelengths through distinct photoreceptors. These include white collar proteins and cryptochromes for blue light, opsins for green light, and phytochromes for red light. By perceiving light as an informational signal, these fungi adapt their growth and developmental processes to environmental conditions (Yu and Fischer, 2019). However, despite these significant insights, the molecular mechanisms behind light perception and signal transduction in basidiomycetes, such as *H. erinaceus*, remain poorly understood. Therefore, this study aims to explore how *H. erinaceus* responds to light at the molecular level, particularly focusing on the proteomic changes induced by exposure to various light wavelengths.

Proteomics offers a powerful tool for investigating the global protein expression profiles of organisms under varying environmental stimuli (Nesatyy and Suter, 2007). By applying techniques such as liquid chromatography-tandem mass spectrometry (LC-MS/MS), it is possible to identify differentially expressed proteins (DEPs) in response to specific light wavelengths and to elucidate their roles in regulatory pathways (Aebersold and Mann, 2003; 2016). Previous studies in other fungal species have demonstrated that light-regulated proteins are involved in stress response, energy metabolism, and biosynthetic processes (Yu and Fischer, 2019). For instance, Park and Jang (2020) investigated the proteomic response of *Lentinula edodes* to blue light, revealing significant changes in protein expression associated with energy metabolism and cell wall formation. Their findings showed that blue light exposure led to an increase in pileus size and thickness, as well as changes in stipe development. Additionally, their analysis highlighted the upregulation of proteins linked to energy metabolism and cell wall synthesis, while proteins involved in other cellular functions were downregulated. These results underscore the vital role of blue light in shaping the physiological and biochemical pathways of *L. edodes*, suggesting its potential to enhance mushroom cultivation and improve yield and quality. Despite these advancements, no comprehensive proteomic study has been conducted to evaluate how *H. erinaceus* responds to light at the protein level. Therefore, this study aims to investigate the light-induced proteomic changes in *H. erinaceus* mycelia exposed to different light wavelengths.

## 2 Materials and methods

### 2.1 Mushroom strain preparation

The *H. erinaceus* strain used in this study was isolated from fresh fruiting bodies cultivated at Thammasat University (Pathum Thani, Thailand). Tissue fragments were aseptically excised from the inner portion of the fruiting body and transferred onto potato dextrose agar (PDA) plates. Cultures were incubated at 25 ± 1 °C until the mycelium fully colonized the agar surface.

### 2.2 Preparation of PDA using commercial medium

PDA medium was prepared using commercially available dehydrated PDA powder (HiMedia, Maharashtra, India). The powder was dissolved in distilled water at a concentration of 39 g/L, following the manufacturer’s instructions. The mixture was heated with constant stirring until it was completely dissolved and then sterilized by autoclaving at 121 °C for 15 minutes. After autoclaving, the medium was allowed to cool to approximately 50 °C and poured into sterile petri dishes under aseptic conditions. After solidification, actively growing mycelial margins obtained from Section 1 were excised into small fragments (1 × 1 cm) and subcultured onto fresh PDA plates. The inoculated cultures were then incubated at 25 ± 1 °C under either dark or controlled light conditions for 3 days prior to LED light treatments.

### 2.3 Experimental design and light treatments of *H. erinaceus*

This experiment was designed to investigate the effect of different light spectra on the mycelial growth of *H. erinaceus*. The experiment consisted of 4 light treatments - blue light (BL; wavelength 450 nm), green light (GR; wavelength 520 nm), red light (RD; wavelength 660 nm), and a combination of red, green, and blue light (RGB) (**Supplementary Figure 1**) - along with a control treatment (CT; darkness) where the cultures were maintained in complete darkness. The light intensity for all treatments was maintained at 40 ± 5 µmol m^-²^s^-¹^, with a daily exposure period of 8 hours. Following this, the inoculated substrates were incubated under the designated light conditions at a temperature of 25 ± 1 °C. The light sources used were LED panels, each calibrated to ensure uniform light distribution across the incubation area. The spectral characteristics and intensity of each light source were verified using the light spectrum analyzer (UPRtek PG200N, Miaoli, Taiwan).

The mycelium of *H. erinaceus* was cultured under the designated light test conditions for 15 days to obtain sufficient biomass. At the end of the cultivation period, mycelia were aseptically harvested by gently scraping the surface of the solid medium. The collected biomass was then frozen at -80 °C and stored until further analysis.

### 2.4 Proteomic analysis using a non-targeted label-free approach

#### 2.4.1 Protein extraction

The samples were resuspended in 200 µl of lysis buffer (2 M thiourea, 7 M urea, 4% CHAPS, and 1% protease inhibitor cocktail). The sample cells were disrupted by sonication on ice using a Vibra-Cell VCX750 sonicator (Fisher Scientific, Massachusetts, USA) operated at an amplitude of 60 with 0.5 duty cycles. Then, samples were centrifuged at 14,000 rpm for 30 min at 4 °C for cell debris removal. Supernatant was collected and stored at -80 °C until further use (Aponte et al., 2009).

#### 2.4.2 In-solution digestion

Protein samples were purified using a commercial clean-up kit (GE Healthcare, USA). The resulting protein pellets were dissolved in 8 M urea, and protein concentrations were determined using the Bradford assay (Bio-Rad Protein Assay, Bio-Rad Laboratories, CA, USA). For each sample, 30 μg of protein was reduced in reduction buffer containing 100 mM dithiothreitol in 100 mM triethylammonium bicarbonate (TEAB) at 37 °C for 30 min. Alkylation was subsequently performed by adding 100 mM iodoacetamide in 100 mM TEAB, followed by incubation at room temperature in the dark for 30 min. Excess iodoacetamide was quenched by incubation with reduction buffer at room temperature for an additional 15 min. Proteins were then digested with sequencing-grade trypsin (Trypsin Gold, Promega, USA) at 37 °C for 16 h. The resulting peptide mixtures were dried using a nitrogen evaporator (Organomation, USA), resuspended in 0.1% formic acid (FA), and desalted using C18 ZipTips. After desalting, peptides were again dried under nitrogen and stored at -80 °C until further analysis. Prior to LC-MS/MS analysis, peptides were resuspended in 0.1% FA, and peptide concentrations were measured using a NanoDrop 1000 spectrophotometer (Thermo Fisher Scientific, Bremen, Germany) (Wiśniewski et al., 2009).

#### 2.4.3 Nano-LC-MS/MS analysis

Peptides were analyzed on an LC-MS/MS system including a Nano-liquid chromatograph (Dionex Ultimate 3000, RSLCnano System, Thermo Scientific) in combination with a CaptiveSpray source/Quadrupole ion trap mass spectrometer (Q-ToF Compact, Bruker, Germany). One microgram of peptides was enriched by nano trap column (Acclaim PepMap 100 C18, 100 μm i.d. × 2 cm, 5 μm particle size, 100 Å pore size) and subsequently separated on an analytical column (PepMap 100 C18, 75 μm i.d. × 500 mm, 3 μm particle size). Elution was performed using a linear gradient of 2-95% solvent B over 120 min at a flow rate of 300 nL/min and a column temperature of 60 °C. There were 2 mobile phases: A, 0.1% FA in water, and B, 0.08% FA in 80% acetonitrile. The loading pump solvent consisted of 0.05% TFA in 2% acetonitrile. A gradient of mobile phase B was used as follows: 2% for 5 min, ramped to 35% for 80 min, then ramped to 55% for 20 min, and ramped to 95% for 10 min, then ramped down to 2% for 1 sec and re-equilibrated for 5 min. Drying gas flow and temperature were 5 L/min and 150 °C, respectively, and nebulizer gas pressure was 0.2 bars. MS acquisition rate was 6 Hz, and a positive ionization mode was used with a survey scan mass range of m/z 150-2200. AutoMSn CID fragmentation experiments were performed at low (4 Hz) and high (16 Hz) mass spectral rates for the top 2 most intense precursor ions using 3 sec dynamic exclusion.

#### 2.4.4 MS data processing and statistical analysis

For the discovery phase, raw MS data were processed with MaxQuant software (version 1.6.2.10) coupled with its built-in search engine, Andromeda, for protein identification. The default setting, with the Hericiaceae database downloaded from https://www.uniprot.org, was set to 1 as a label-free approach. Parameter settings used the defaults except the following. Oxidation of methionine and acetylation of the N-terminus were set as variable modifications, whilst carbamidomethyl modification of cysteine was set as a fixed modification. Bruker Q-TOF was selected as the instrument type, with peptide tolerance for first and second searches set as 0.8 and 0.4, respectively. Trypsin/P was set for the identification of peptides with a maximum of two missed cleavages. False discovery rate (FDR) was set at 1% at the peptide level. TOF MS/MS match tolerance was set at 0.8 Da with label-free quantification. A match between run options in the software was used for mass and retention times recalibration between runs.

### 2.5 Bioinformatic analysis

Proteins identified in the previous section were subjected to bioinformatic and statistical analyses. Data visualization was performed using several approaches. Venn diagrams were generated according to the method described by Heberle et al. (2015) to illustrate the overlap of identified proteins among experimental groups. Correlation analysis was conducted and visualized using the corrplot package (Version 0.95) in R. Principal component analysis (PCA) was performed in R to assess overall proteomic variation among samples, and PCA plots were generated using the ggplot2 package (version 4.0.1). Heatmap visualization was carried out using the pheatmap package (Version 10.0.13) in R, following the method described by Kolde (2025). Differentially expressed proteins were identified using the limma package (Version 3.58.1) in R, as described by Ritchie et al. (2015), and the results were visualized using volcano plots generated with the ggplot2 package (version 4.0.1) (Wickham, 2016). Proteins with log_2_ fold change ≥ 1 and adjusted p-value < 0.05 were considered significantly differentially expressed. Gene Ontology (GO) enrichment analysis was performed using the clusterProfiler package (Version 4.10.1) in R, following the methodology of Yu et al. (2012), in accordance with the guidelines and annotations provided by the Gene Ontology database (https://geneontology.org/) and UniProt (https://www.uniprot.org/). Functional classification and metabolic pathway analysis of differentially abundant proteins were conducted using the Kyoto Encyclopedia of Genes and Genomes (KEGG) database (Aramaki et al., 2020). The number of differentially expressed proteins assigned to each functional category was quantified, and enrichment significance was evaluated using a hypergeometric distribution test. In addition, annotation of carbohydrate-active enzyme (CAZyme) families was performed using the dbCAN3 database as described by Zheng et al. (2023).

## 3 Results

### 3.1 Morphological response of *H. erinaceus* mycelium to different light treatments

**Figure 1:**
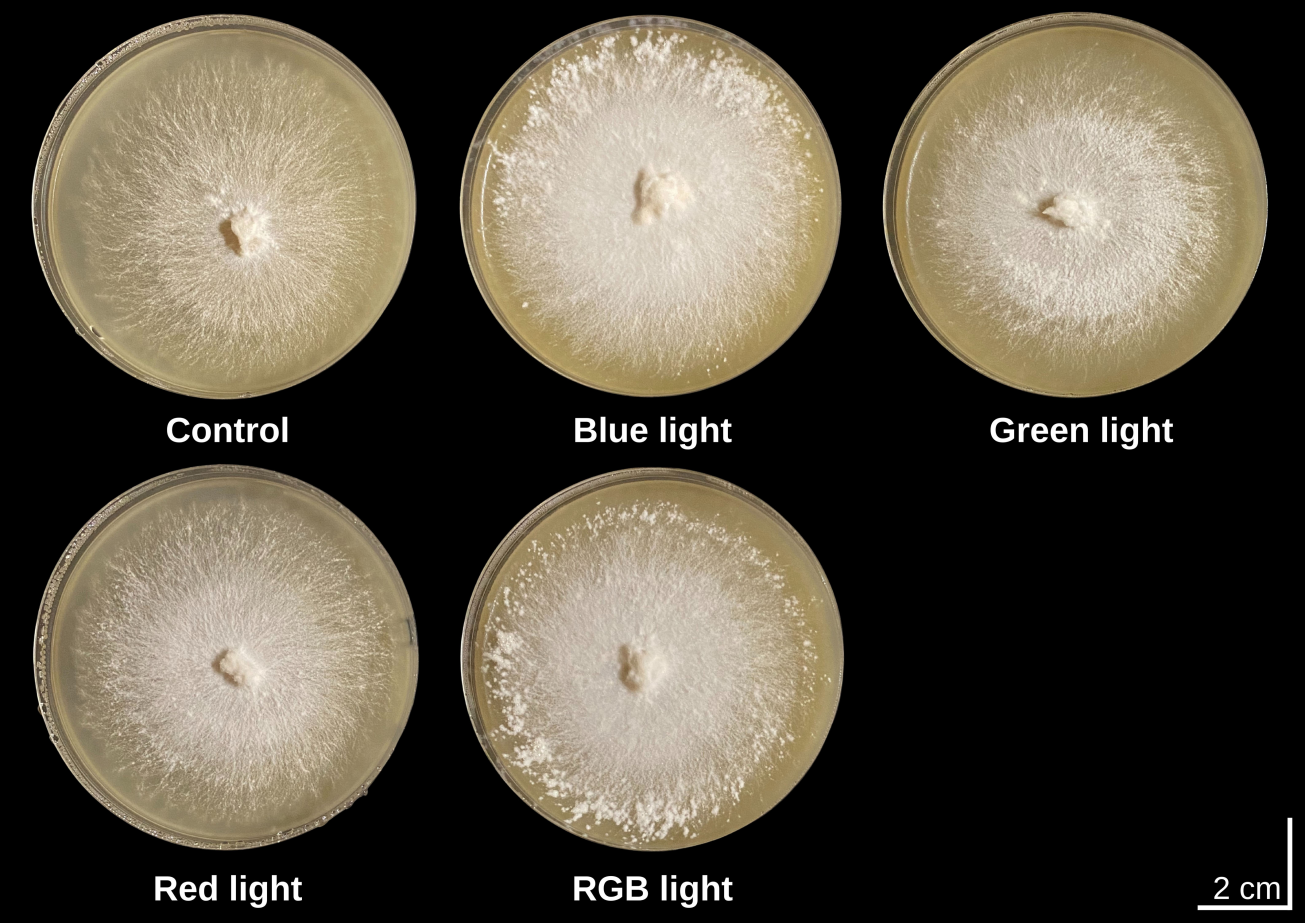
Colony morphology of *H. erinaceus* mycelium at 15 days of incubation under different light treatments.

After 15 days of incubation, *H. erinaceus* mycelium exhibited characteristic colony morphologies under different light treatments: control, blue, green, red, and RGB light (**Figure 1**). In the control treatment, colonies were circular with a dense, bright-white surface and a slightly elevated central region from which hyphae extended radially, representing the characteristic growth pattern of unstimulated mycelia. Exposure to blue light produced uniformly white colonies with abundant aerial hyphae aggregates in a highly symmetrical, radiating pattern, suggesting that blue wavelengths promote vigorous vertical hyphal development. Under green light, colonies remained circular but exhibited a compact, well-defined central zone and more finely distributed peripheral hyphae, while maintaining a stable white coloration. Red light resulted in smooth, compact colonies with clearly delineated margins and a uniform white surface, indicating steady but less aerially pronounced growth compared to blue light. In contrast, RGB light induced dense, cotton-like mycelia with bright white pigmentation and evenly radiating hyphae originating from the inoculation point, with discrete hyphal aggregates observed across the colony surface, reflecting a potential synergistic effect of multiple wavelengths on hyphal expansion. Across all treatments, colonies retained a circular growth habit, and no pigmentation other than white was observed during the 15-day cultivation period, indicating that light quality influenced colony architecture.

### 3.2 Proteomics statistics of *H. erinaceus* samples for growth in different light qualities and the correlation among the five groups of samples

**Figure 2:**
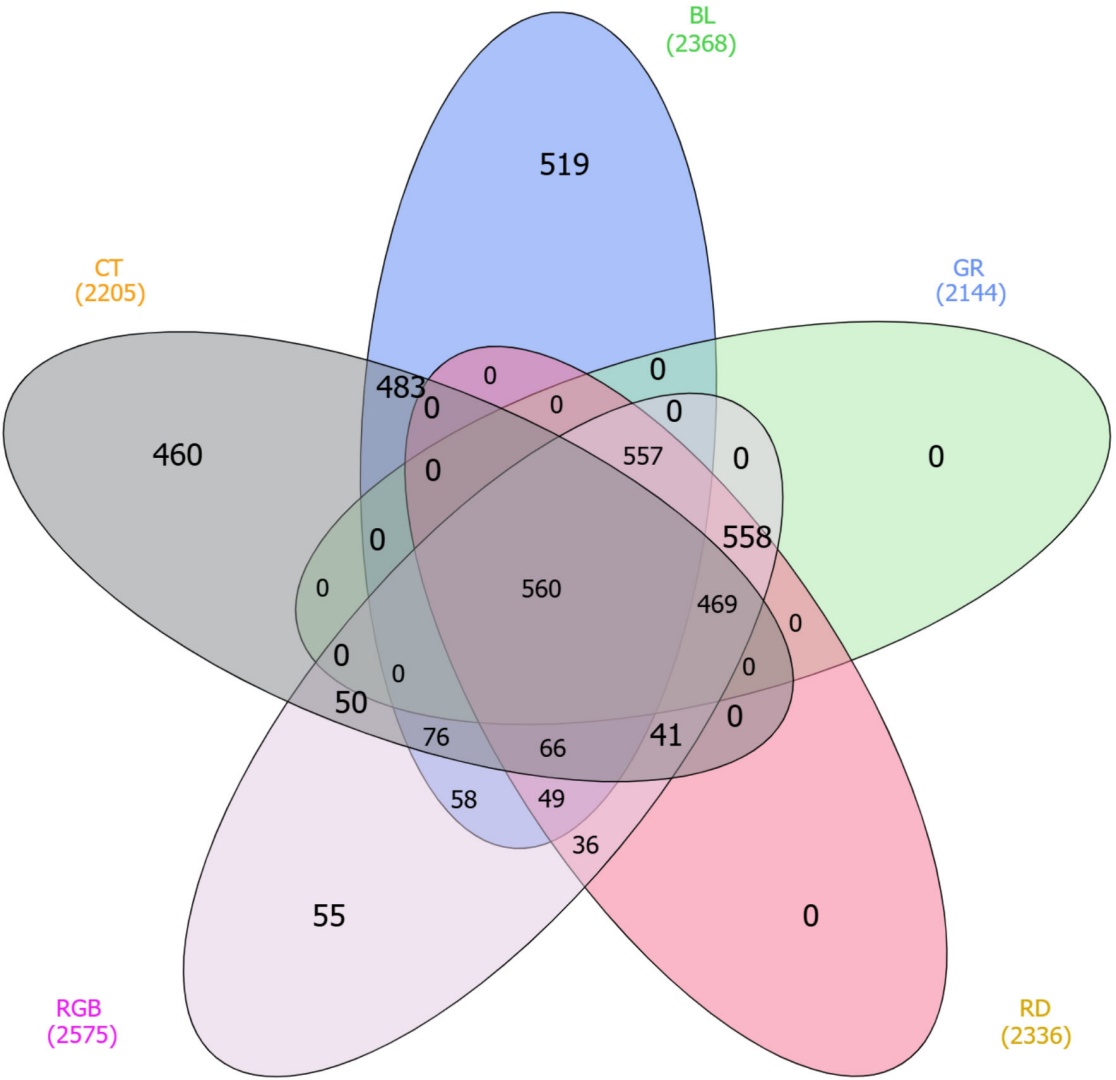
Venn diagram showing the distribution of identified proteins in *H. erinaceus* mycelium after 18 days of cultivation under different light treatments: control (CT), blue (BL), green (GR), red (RD), and RGB. Overlapping regions indicate shared proteins, while non-overlapping sections represent condition-specific proteins.

A label-free LC-MS/MS quantitative proteomic analysis was applied to examine the protein expression profiles of *H. erinaceus* mycelia cultured for 15 days under various light spectra during the later cultivation stage. Five treatment groups, each including three replicates, were analyzed, resulting in the identification of 4,618 proteins using the *H. erinaceus* reference proteome database. Comparative proteomic profiling was then performed to assess the influence of blue (BL), green (GR), red (RD), and combinations of red, green, and blue (RGB) light on *H. erinaceus* mycelium in comparison with the control (CT) treatment (**Figure 2**). Protein identifications totaled 2,205 in CT, 2,368 in BL, 2,144 in GR, 2,336 in RD, and 2,575 in RGB treatments. Among these, 560 proteins were consistently detected in all experimental groups, representing the conserved core proteome associated with the vegetative growth phase of *H. erinaceus*. The next largest subset comprised 558 proteins detected in all light-treated groups but absent in the control. Distinct subsets of proteins were uniquely expressed under specific light treatments, suggesting light-dependent regulation of protein synthesis. The blue-light treatment revealed 519 unique proteins, suggesting light-responsive mechanisms potentially associated with photoreceptor activation and cellular adaptation. The control and red-light treatments revealed 460 and 55 unique proteins, respectively, corresponding to basal and red-light–specific metabolic processes. The green-light treatment did not show any uniquely detected proteins, suggesting extensive overlap with other spectral conditions. The RGB treatment, which included combined wavelengths, yielded 55 unique proteins, potentially reflecting integrative responses to broad-spectrum illumination. Intermediate overlaps among treatments ranged between 36 and 76 shared proteins, reflecting partial convergence of proteomic responses across specific wavelengths. Collectively, these findings indicate that while *H. erinaceus* maintains a stable core proteome independent of illumination; distinct light spectra induce specific protein expression patterns.

**Figure 3:**
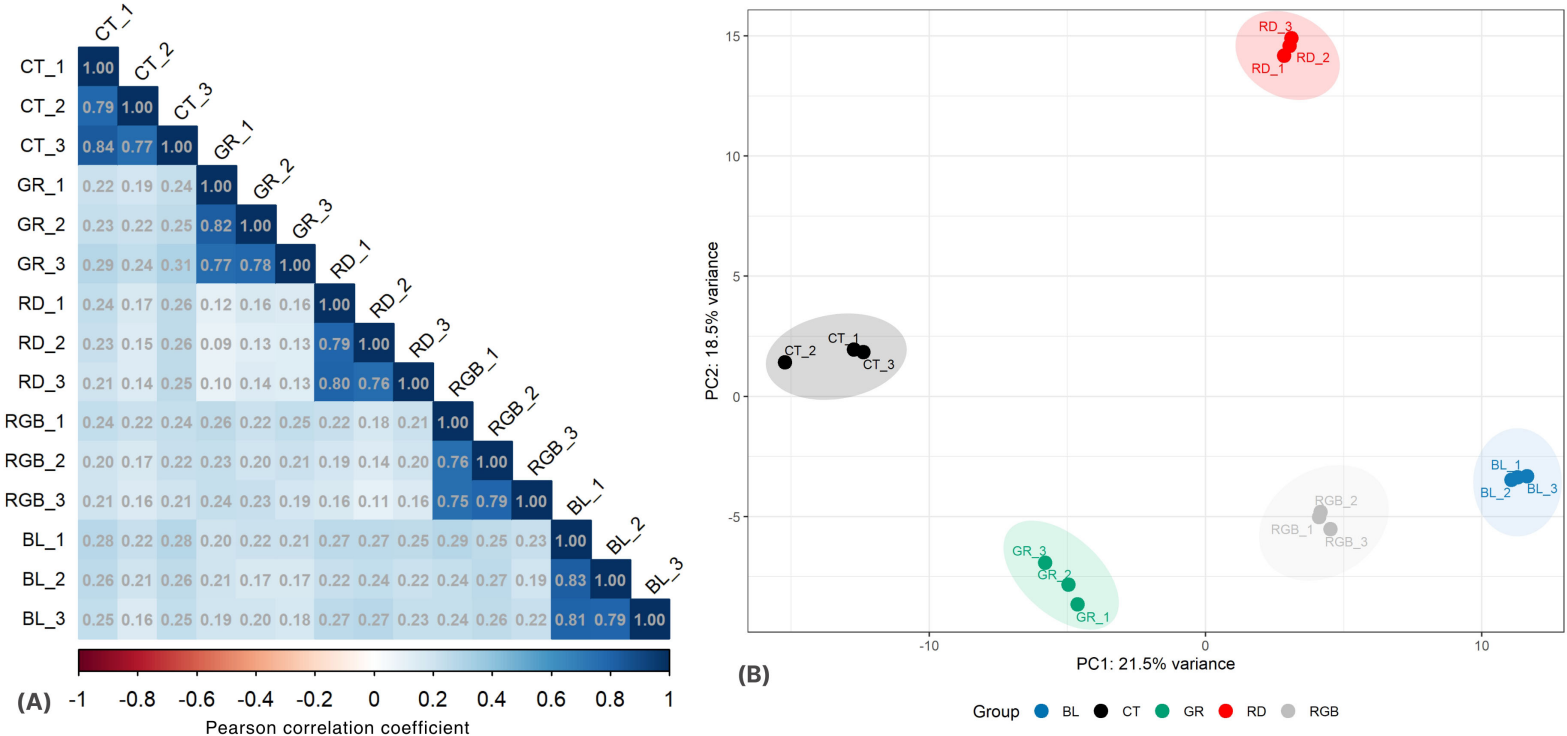
Correlation analysis and cluster analysis of the proteomic dataset of *H. erinaceus* mycelial proteomes under different light treatments. **(A)** Pairwise Pearson correlation coefficient (PCC) heatmap showing the correlation among proteome data. **(B)** Principal component analysis (PCA) of proteome data from the five groups of samples.

To evaluate the reproducibility and consistency of proteomic data across biological replicates, Pearson correlation coefficient (PCC) analysis was performed for all samples obtained under different light treatments. The pairwise correlation matrix revealed strong intra-group consistency, confirming the reliability of the label-free LC-MS/MS quantification (**Figure 3A**). The three control replicates exhibited exceptionally high correlation values (PCC = 0.94-1.00), indicating robust reproducibility within the untreated samples. Similarly, the blue-light treatment showed strong internal consistency (PCC = 0.90-0.93), followed by the red-light treatment (PCC = 0.87-0.89), and the green-light treatment (PCC = 0.89-0.93). The RGB treatment also demonstrated acceptable reproducibility, with intra-group correlations ranging from 0.87 to 0.88. By contrast, inter-group correlations between different light treatments were comparatively lower (PCC = 0.10-0.27), suggesting that distinct light wavelengths induced substantial differences in the proteomic profiles of *H. erinaceus* mycelia. These findings validate the consistency of biological replicates within each treatment group while indicating that light quality exerts measurable effects on overall protein expression patterns.

Principal component analysis (PCA) was performed to evaluate the overall variance and clustering patterns of the proteomic profiles of *H. erinaceus* mycelia cultivated under different light treatments. The first two principal components (PC1 and PC2) accounted for 21.5% and 18.5% of the total variance, respectively (**Figure 3B**). The PCA score plot revealed clear separation among the five experimental groups, indicating distinct proteomic responses to the different light spectra. The three replicates within each group clustered closely together, demonstrating strong reproducibility and consistency of the proteomic data. The control samples formed a compact cluster positioned on the negative side of PC1, whereas blue light-treated samples appeared distinctly on the positive side of PC1, reflecting significant alterations in protein expression relative to the control. The green-light treatment was positioned on the lower left quadrant, while the red-light treatment appeared in the upper right quadrant, each forming discrete clusters. The RGB treatment occupied a central but separate position between the blue and green clusters, suggesting an intermediate proteomic profile that integrates responses to multiple wavelengths. The clear group separation along both principal components indicates that different light qualities exerted marked effects on the global proteomic landscape of *H. erinaceus* mycelium. This result further supports the hypothesis that wavelength-specific illumination regulates distinct metabolic and cellular pathways associated with the organism’s vegetative growth phase.

**Figure 4:**
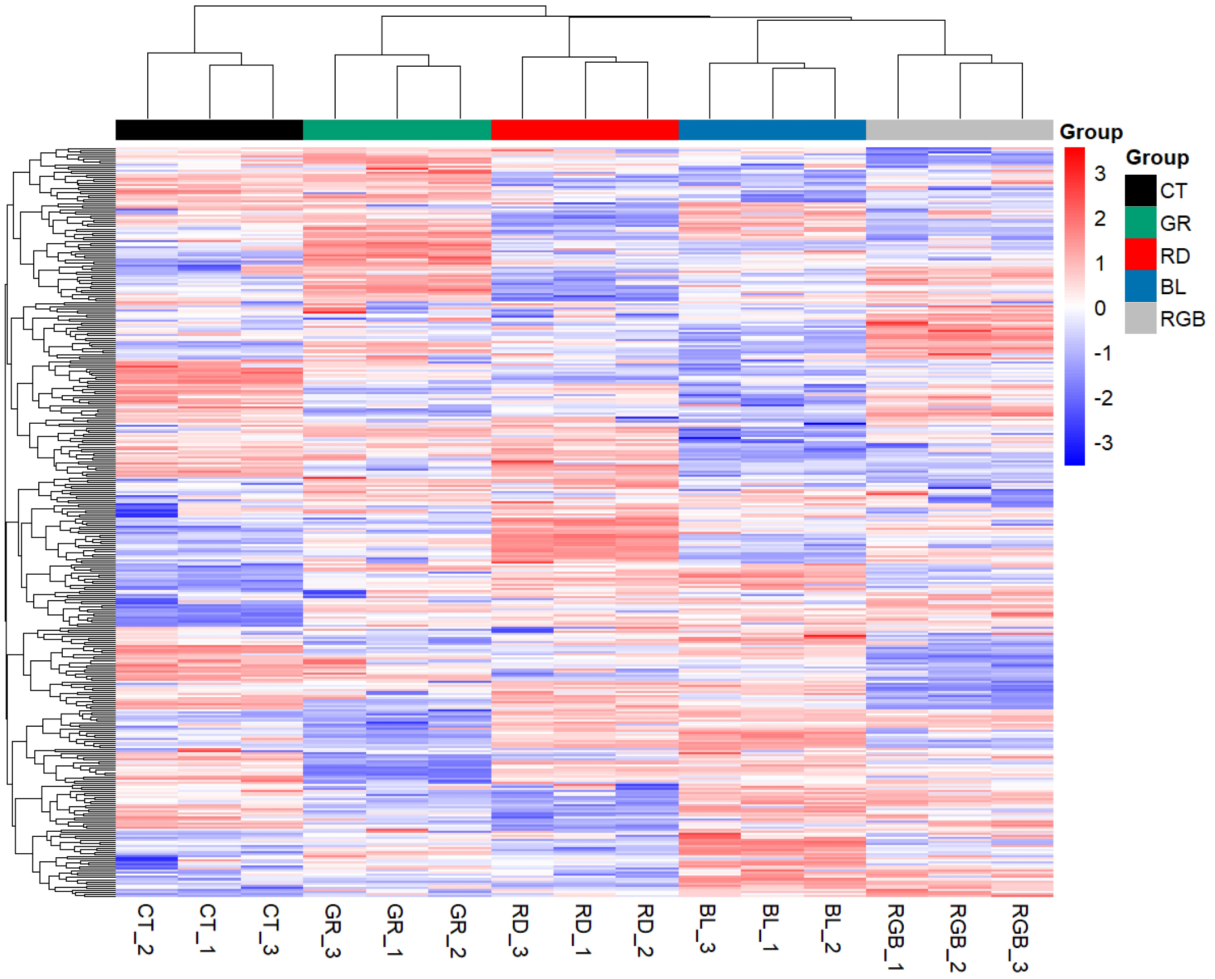
Hierarchical clustering heatmap of all quantified proteins in *H. erinaceus* mycelium cultured for 18 days under different light treatments. The color scale represents relative protein expression levels (red = upregulated; blue = downregulated). Clustering was based on Euclidean distance and the complete linkage method.

A hierarchical clustering heatmap was generated to visualize the global expression patterns of all quantified proteins from *H. erinaceus* mycelia cultivated under different light conditions (**Figure 4**). The clustering analysis revealed clear differentiation in protein expression profiles among the treatment groups, demonstrating that light wavelength exerts a strong regulatory effect on the proteomic composition of *H. erinaceus* mycelia. The control samples displayed a distinct expression pattern that was clearly separated from all illuminated groups, confirming that exposure to light induces substantial proteomic remodeling and physiological adaptation in the fungus. Among the light-treated samples, blue-light and RGB conditions were grouped in close proximity, indicating shared regulatory responses and partially overlapping signaling mechanisms. This resemblance suggests that both short-wavelength (blue) and combined-spectrum (RGB) light may trigger similar photoreceptor-dependent pathways, thereby coordinating the regulation of oxidative stress responses, carbohydrate metabolism, and secondary metabolite production. In contrast, the red-light and green-light treatments formed distinct clusters, each characterized by unique sets of differentially expressed proteins, signifying wavelength-specific modulation of metabolic activity and developmental processes within *H. erinaceus* mycelia.

### 3.3 Identification of differentially expressed proteins

**Figure 5:**
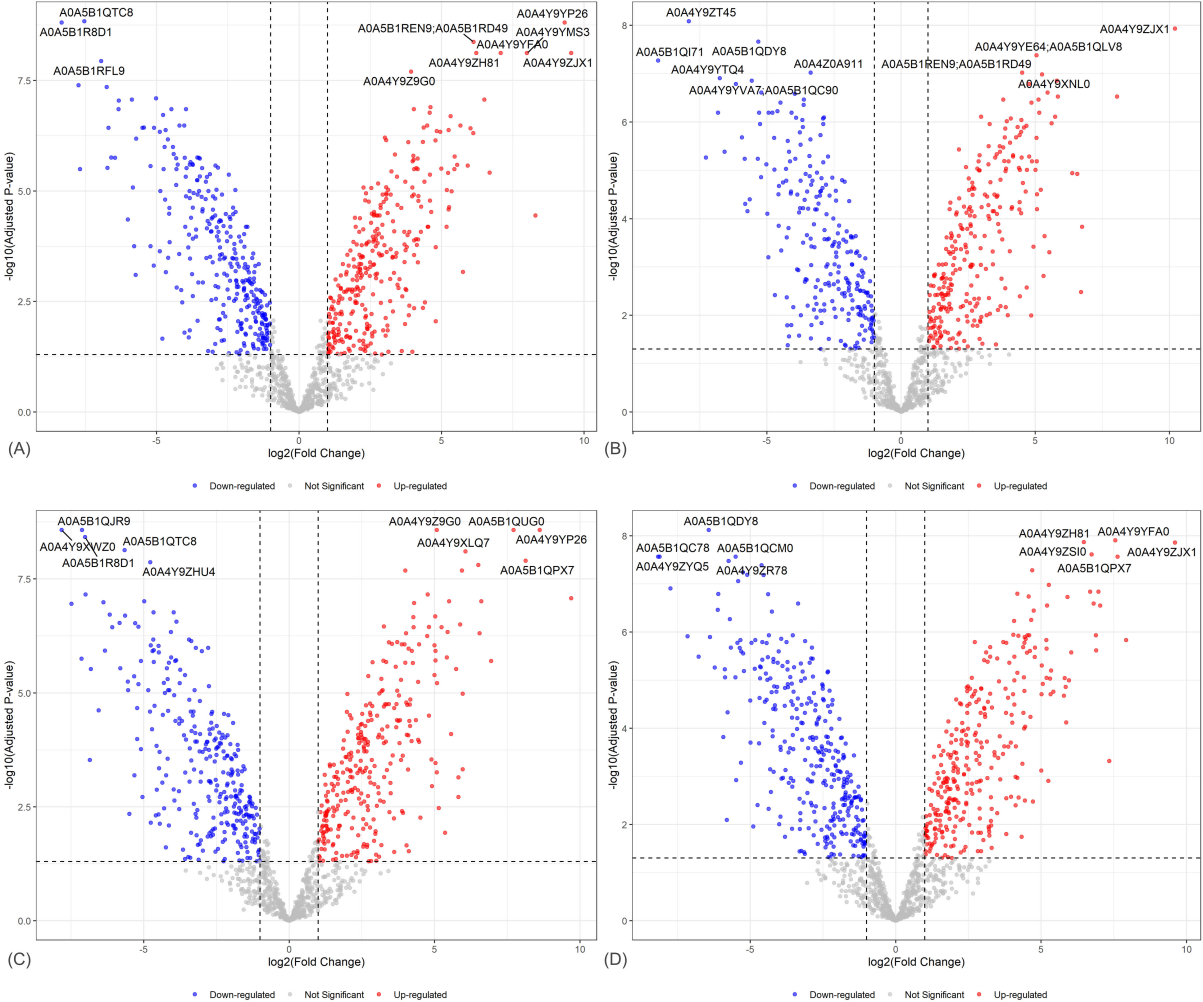
Volcano plots of differentially expressed proteins (DEPs) in *H. erinaceus* mycelia under different light conditions. Volcano plots illustrate proteins that are up- and down-regulated in the mycelia of *H. erinaceus* exposed to light compared with the control group, where **(A)** represents BL vs CT, **(B)** GR vs CT, **(C)** RD vs CT, and **(D)** RGB vs CT.

The volcano plots (**Figures 5A–D**) depict the overall distribution of DEPs in *H. erinaceus* mycelia subjected to blue, green, red, and RGB light treatments compared with the control treatment. Each plot represents the log_2_ fold change versus the log_10_ adjusted p-value, with significantly up-regulated proteins marked in red and down-regulated proteins in blue. Differential proteomic profiling revealed that light of different wavelengths induced distinct yet partially overlapping expression patterns. Among these, red and blue light triggered the most extensive proteomic alterations, characterized by a higher abundance of significantly up-regulated proteins such as A0A4Y9YP26, A0A4Y9ZJX1, and A0A4Y9YZH81, which are likely involved in photoreceptor-mediated signaling and oxidative stress defense. In contrast, proteins including A0A5B1RFL9 and A0A5B1QTC8 were consistently down-regulated, suggesting attenuation of dark-adaptive or energy-storage pathways. Green and RGB light exposures produced moderate but distinct changes in protein expression, with up-regulated proteins such as A0A5B1QPX7 and A0A4Y9P26 indicating partial activation of shared light-responsive mechanisms (**Supplementary Tables 1**-**4**). Collectively, these results indicate that *H. erinaceus* undergoes pronounced, wavelength-dependent proteomic remodeling, with red and blue light exerting the greatest regulatory influence, underscoring their pivotal roles in fungal photomorphogenesis and adaptive metabolic regulation.

**Figure 6:**
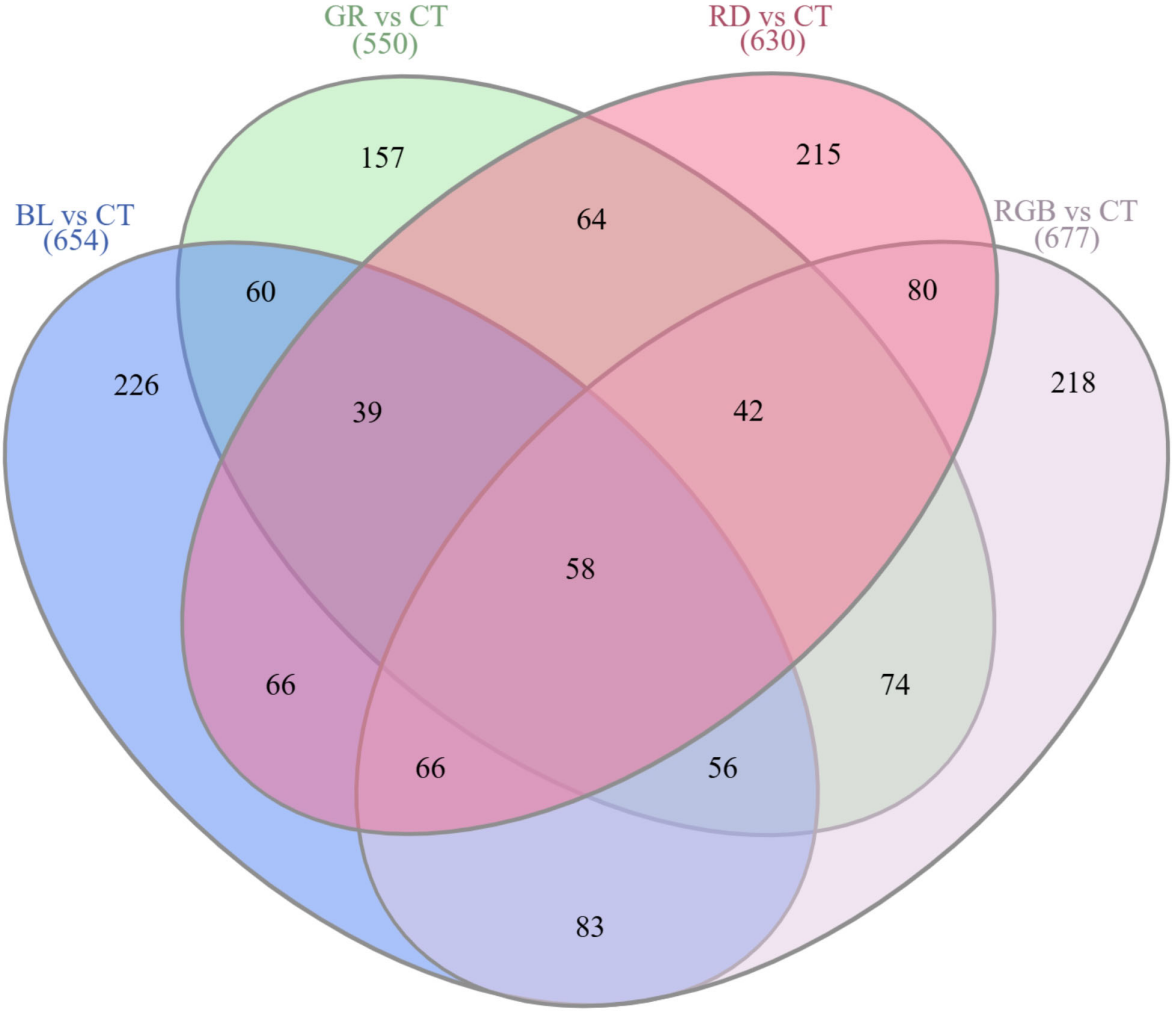
Venn diagram illustrating the overlap of DEPs identified among the four light treatments compared to the control, highlighting both shared and wavelength-specific protein expression patterns.

The Venn diagram illustrates the distribution and overlaps of DEPs detected under various light treatments in *H. erinaceus* (**Figure 6**). Comparative proteomic analysis relative to the control treatment revealed both wavelength-specific and shared responses, with 654, 550, 630, and 677 DEPs identified under blue, green, red, and RGB light, respectively. A core set of 58 DEPs was commonly regulated across all treatments, indicating the presence of fundamental light-responsive mechanisms in *H. erinaceus*. Each light condition also induced a distinct proteomic signature, most notably under blue (226 unique DEPs), RGB (218 unique DEPs), and red light (215 unique DEPs). These findings suggest that blue light predominantly reprograms metabolic and defense-related pathways, red light enhances energy metabolism and secondary metabolite biosynthesis, and RGB light integrates multiple spectral cues for complex proteomic regulation. Collectively, these results demonstrate that *H. erinaceus* exhibits both wavelength-dependent and conserved proteomic adaptations, reflecting a highly coordinated photobiological response system.

### 3.4 Gene ontology analysis for the DEPs in each comparison to biological process, cellular component, and molecular function categories

**Figure 7:**
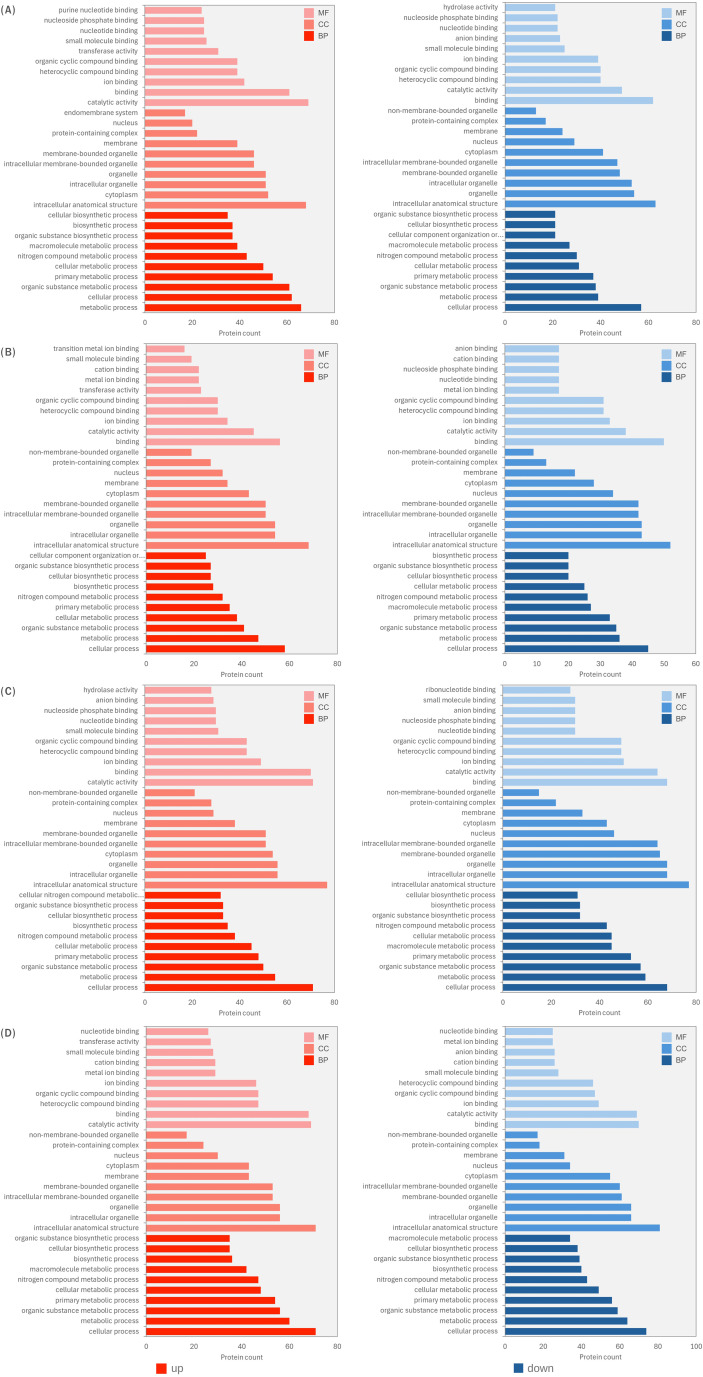
Top 10 Gene Ontology (GO) enrichment analysis of significantly up- and down-regulated DEPs in response to different light treatments compared with the control (p < 0.05): **(A)** BL vs CT, **(B)** GR vs CT, **(C)** RD vs CT, and **(D)** RGB vs CT.

Gene Ontology (GO) enrichment analysis was performed to characterize the functional profiles of DEPs in *H. erinaceus* mycelia exposed to four light conditions: blue, green, red, and RGB relative to the control treatment (**Supplementary Tables 5**-**8**). The DEPs were categorized into Biological Process (BP), Cellular Component (CC), and Molecular Function (MF), and the top 10 enriched GO terms were evaluated for up-regulated and down-regulated proteins for each treatment. Overall, the GO patterns reveal that light exposure differentially modulates metabolic processes, catalytic activities, and intracellular component organization, suggesting distinct physiological adjustments specific to each wavelength. Blue light predominantly up-regulated proteins associated with metabolic process and organic substance metabolic process, which represented the most enriched BP categories, followed by protein metabolic process, organonitrogen compound biosynthetic process, amide metabolic/biosynthetic process, peptide metabolic/biosynthetic process, and carbohydrate derivative biosynthetic process. In the CC category, the most enriched terms were cytoplasm (highest protein count), endomembrane system, endoplasmic reticulum, and membrane protein complex, indicating strong activation of cytoplasmic and endomembrane-associated activities. For MF, enrichment was dominated by binding, particularly ion binding, metal ion binding, transition metal ion binding, and transferase activity, reflecting enhanced enzyme-mediated metabolism. Conversely, down-regulated DEPs were mainly enriched in BP terms including cellular process, macromolecule metabolic process, transport, establishment of localization, proteolysis, and cellular component organization. Down-regulated CC terms were centered on intracellular anatomical structure, organelle, membrane-bounded organelle, and nucleus, while MF terms were dominated by protein binding, adenyl nucleotide binding, and hydrolase activity, suggesting partial suppression of nucleotide-dependent and proteolytic functions (**Figure 7A**). Under green light, up-regulated proteins were enriched in BP categories related to cellular component organization or biogenesis, organelle organization, regulation of gene expression, regulation of macromolecule metabolic process, and regulation of biosynthetic process, indicating a stronger emphasis on structural organization and regulatory processes compared to blue light. CC enrichment included intracellular anatomical structure (highest), nucleus, and Golgi apparatus, suggesting enhanced intracellular structural remodeling. In MF, the most enriched terms were transferase activity, transition metal ion binding, zinc ion binding, and acyltransferase activity. Down-regulated proteins were enriched in MF terms such as anion binding, nucleotide binding, ribonucleotide binding, and ion binding, while CC terms were dominated by intracellular membrane-bounded organelle, organelle, and membrane-bounded organelle. BP categories such as macromolecule metabolic process, transport, and establishment of localization were reduced, indicating attenuation of membrane-associated metabolic and trafficking processes (**Figure 7B**). Red light induced strong up-regulation of BP terms including cellular process (highest enrichment), cellular component biogenesis, ncRNA metabolic process, nucleobase-containing small molecule metabolic process, cell cycle, and chromosome segregation, suggesting enhanced cell cycle progression and nucleic acid metabolism. CC enrichment was dominated by intracellular anatomical structure, cytoplasm, and membrane protein complex, while MF terms prominently included ion binding, small molecule binding, nucleotide binding, nucleoside phosphate binding, purine ribonucleoside triphosphate binding, and GTP binding, indicating activation of nucleotide-dependent regulatory proteins. Down-regulated DEPs were enriched in BP categories such as macromolecule metabolic process, transport, proteolysis, and DNA repair, with CC terms including organelle, intracellular membrane-bounded organelle, and membrane-bounded organelle. MF down-regulation was associated with ion binding, heterocyclic compound binding, and organic cyclic compound binding, reflecting selective suppression of specific substrate-binding activities (**Figure 7C**). RGB treatment produced the broadest transcriptional impact. Up-regulated BP terms were strongly enriched in cellular process (highest), cellular nitrogen compound biosynthetic process, aromatic compound biosynthetic process, heterocycle biosynthetic process, and nucleobase-containing compound biosynthetic process, indicating coordinated activation of nitrogen and nucleic acid-related biosynthesis. CC enrichment included intracellular membrane-bounded organelle, nucleus, and non-membrane-bounded organelle, while MF categories were dominated by binding (highest), metal ion binding, cation binding, transition metal ion binding, and transferase activity. In contrast, down-regulated proteins were primarily associated with small molecule metabolic process, organic acid metabolic process, carboxylic acid metabolic process, and cellular lipid metabolic process, indicating repression of specific primary metabolic branches. CC terms such as intracellular anatomical structure and membrane-bounded organelle were also reduced, alongside MF terms including ion binding and organic cyclic compound binding (**Figure 7D**).

Across all light treatments, metabolic and biosynthetic processes (BP) were consistently enriched in both up- and down-regulated DEPs, demonstrating that illumination serves as a key regulator of primary and secondary metabolism in *H. erinaceus*. Enrichment of intracellular organelles and membrane-bounded compartments (CC) suggests that light triggers reorganization of intracellular trafficking and compartmentalized metabolic activity. MF terms, particularly catalytic activity and small-molecule binding, were strongly represented, indicating wavelength-dependent modulation of enzymatic pathways. Among the treatments, RGB light induced the broadest functional shifts, reflecting synergistic effects of combined wavelengths on growth- and stress-related processes, while red and blue light produced the most pronounced single-wavelength responses, consistent with the known roles of fungal photoreceptors in mediating light-specific regulation.

### 3.5 KEGG pathway enrichment analysis of DEPs

**Figure 8:**
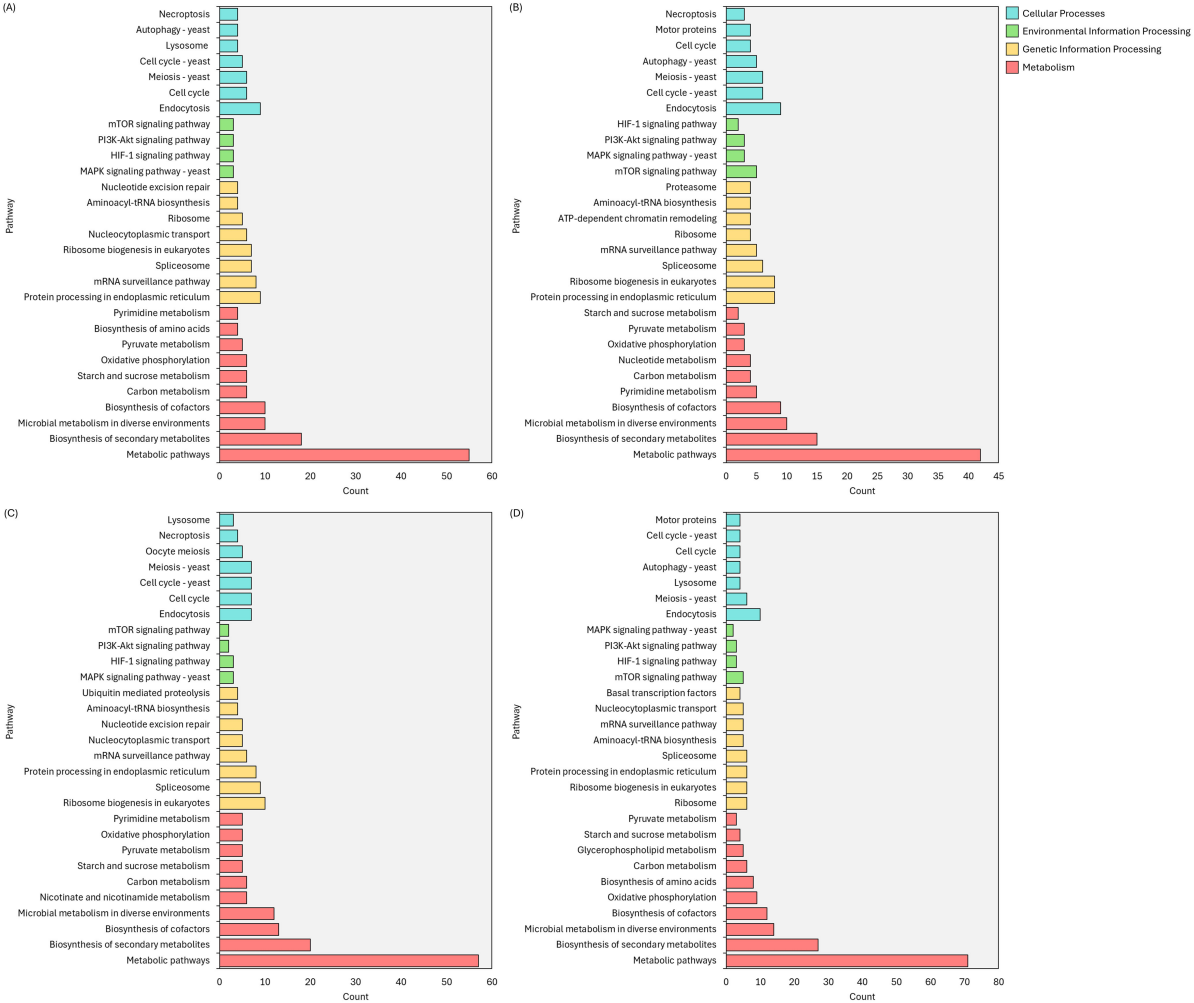
KEGG pathway enrichment of significantly enriched DEPs under different light treatments compared with the control (E-value < 0.05): **(A)** BL vs CT, **(B)** GR vs CT, **(C)** RD vs CT, and **(D)** RGB vs CT.

The KEGG pathway enrichment analysis reveals distinct proteomic responses of *H. erinaceus* mycelium to different light conditions. Across the four treatments, blue, green, red, and RGB DEPs cluster primarily into Metabolism, Genetic Information Processing, Environmental Information Processing, and Cellular Processes, demonstrating that light wavelength strongly influences central metabolic activity and cellular regulation (**Supplementary Tables 9**-**12**). Blue light induced the broadest spectrum of enriched pathways, particularly within metabolic processes. The strong representation of pathways related to carbon metabolism, starch and sucrose metabolism, oxidative phosphorylation, and pyruvate metabolism suggests that blue light markedly elevates energy demand and accelerates carbohydrate catabolism. Concurrent enrichment of ribosome biogenesis, spliceosome function, and ER-associated protein processing further indicates enhanced protein synthesis and post-translational regulation, supported by activation of growth-associated signaling pathways such as mTOR, MAPK, and PI3K-Akt (**Figure 8A**). In comparison, green light elicited a more moderate metabolic response, yet it consistently enriched key biosynthetic and energy-related pathways, including oxidative phosphorylation, aminoacyl-tRNA biosynthesis, and nucleotide metabolism, reflecting sustained but lower-intensity metabolic activity. The presence of chromatin remodeling and ribosome biogenesis suggests ongoing modulation of transcriptional and translational processes, although with weaker activation of major signaling cascades (**Figure 8B**). The red light produced a distinct proteomic signature characterized by selective enrichment of metabolic and regulatory pathways, including carbohydrate metabolism, nicotinate/nicotinamide metabolism, and cofactor biosynthesis, indicating shifts in redox balance and coenzyme utilization. Enhanced enrichment of pathways related to the cell cycle, meiosis, and ubiquitin-mediated proteolysis implies that red light modulates cell division dynamics and protein turnover, while the presence of stress-related pathways such as endocytosis and necroptosis points to the activation of cellular quality-control mechanisms (**Figure 8C**). In contrast to the single-wavelength treatments, RGB light generated the strongest overall metabolic response, showing the highest enrichment of pathways associated with global metabolism, secondary metabolite biosynthesis, and carbon flux. This broad activation included pronounced upregulation of glycerophospholipid metabolism, pyruvate metabolism, and amino acid biosynthesis, consistent with intensified membrane formation and biomass accumulation, alongside substantial enrichment of ribosome biogenesis, ER protein processing, and nucleocytoplasmic transport. Collectively, RGB light also strongly activated major environmental information processing pathways (mTOR, MAPK, PI3K-Akt), indicating a synergistic effect of combined wavelengths on metabolism, protein synthesis, and cellular regulatory networks (**Figure 8D**).

Light wavelength exerts a significant influence on the proteomic landscape of *H. erinaceus* mycelium. Blue and RGB light strongly upregulate core metabolic pathways, secondary metabolite biosynthesis, and protein translation, highlighting their potential for promoting mycelial biomass production and enhancing the synthesis of bioactive compounds. In contrast, red and green light induce more selective metabolic responses: red light predominantly activates stress-responsive and regulatory pathways, whereas green light primarily maintains metabolic homeostasis. Collectively, these findings emphasize the pivotal role of light quality in modulating fungal metabolism and optimizing cultivation strategies.

### 3.6 Identified carbohydrate-active enzymes in the four LED light treatments

**Figure 9:**
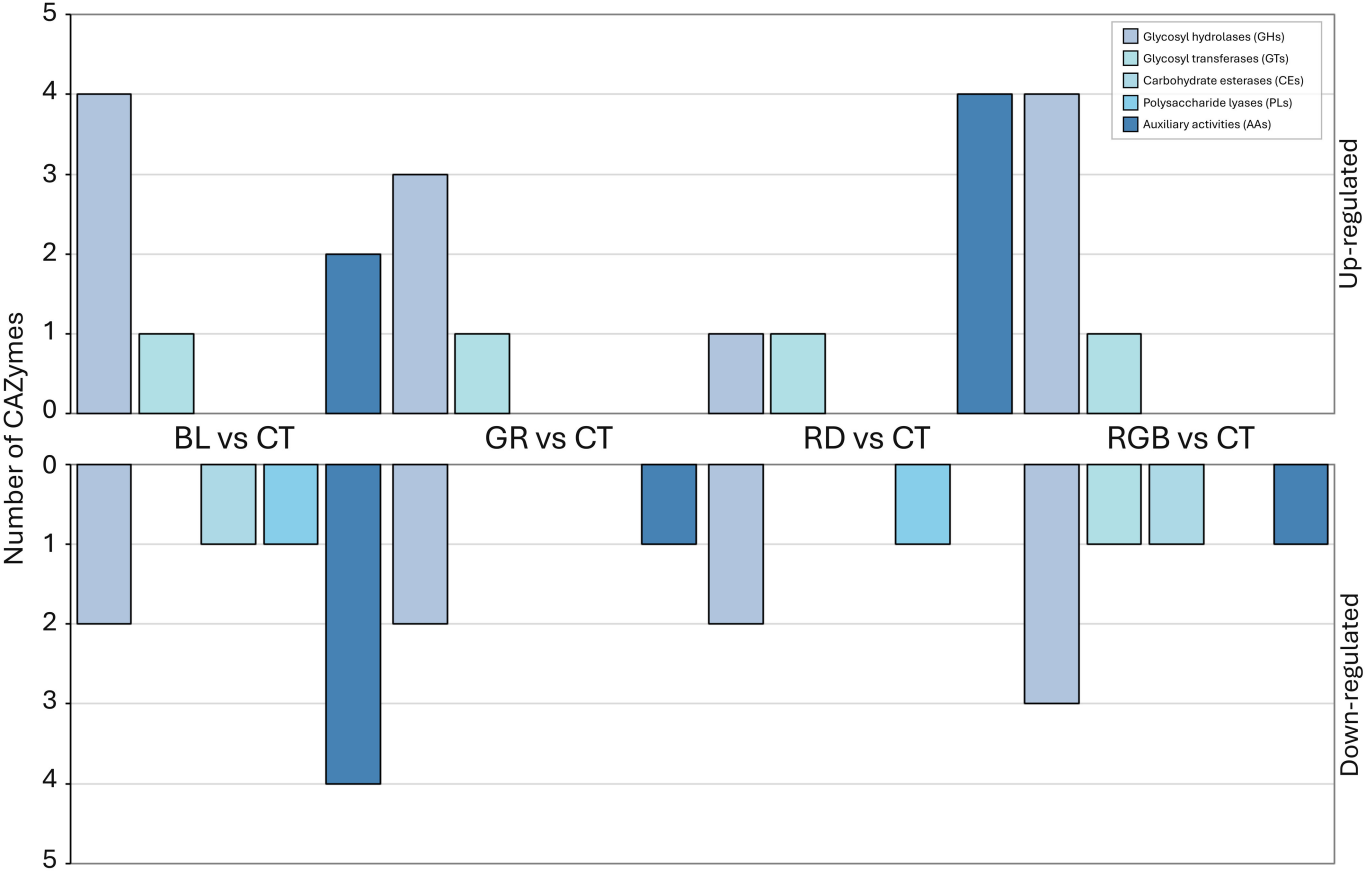
Up- and down-regulated CAZyme families in *H. erinaceus* following exposure to blue, green, red, and RGB LED lighting, showing differential expression across GHs, GTs, CEs, PLs, and AAs.

The comparative analysis of CAZyme expression under the four LED light treatments revealed distinct wavelength-dependent regulatory patterns across major CAZyme families, including glycosyl hydrolases (GHs), glycosyl transferases (GTs), carbohydrate esterases (CEs), polysaccharide lyases (PLs), and auxiliary activity (AA) enzymes in *H. erinaceus* mycelium (**Figure 9**). Under blue light, GH family members were strongly induced, with four GHs: GH47 (A0A4Y9XPD5), GH16_1 (A0A4Y9XW84), GH13_22 (A0A4Y9ZJE0), and GH31_5 (A0A4Z0A5X2) exhibiting significant up-regulation (logFC > 2), while AAs family enzymes were consistently down-regulated. The green light produced a milder regulatory pattern, exhibiting moderate up-regulation of GHs and CEs and down-regulation of selected GHs and GTs. Red light primarily increased AAs enzyme expression, with AA9 (A0A4Y9Y2U6), AA7 (A0A4Y9YB99), AA3_2 (A0A4Y9Z7E8), and AA3_3 (A0A4Z0A14) up-regulated, accompanied by down-regulation of multiple GHs. The RGB treatment generated the most diverse expression profile, featuring GHs up-regulation alongside broad down-regulation across GHs, GTs, CEs, and AAs. Collectively, each wavelength induced a distinct transcriptional profile across CAZyme families, with BL and RGB treatments producing the most pronounced changes.

## 4 Discussion

Light is a critical environmental factor influencing the growth and development of mushrooms (Chiang et al., 2017). It plays a fundamental role in regulating physiological processes, including mycelial expansion and metabolic activity. Variations in light wavelength, intensity, and exposure duration can significantly impact morphology and protein expression (Chutimanukul et al., 2025). This study examined the effects of different light wavelengths on the morphological and proteomic responses of *H. erinaceus*, and the results reveal several wavelength-dependent patterns that advance our understanding of fungal photoregulation. The results demonstrate that light quality functions as a key regulatory cue, driving distinct morphological outcomes across treatments. Despite these differences, colonies consistently retained a circular growth pattern and exhibited no pigmentation other than white throughout the 18-day cultivation period, indicating that light quality influenced colony structure. The morphological distinctions observed under blue, green, red, and RGB light highlight the marked sensitivity of *H. erinaceus* mycelia to specific wavelengths, which appear to modulate hyphal density, aerial hypha formation, and colony compactness in a wavelength-dependent manner. These responses likely represent adaptive mechanisms that enable the fungus to optimize growth, environmental perception, and metabolic activity under varying light environments (Ballario et al., 1998; Malzahn et al., 2010; Olmedo et al., 2013). Notably, light is a key environmental factor influencing protein expression in mushrooms and can generate divergent responses depending on the wavelength of illumination (Pawlik et al., 2019; Ye et al., 2021). Although several transcriptomic studies have examined light-induced regulation in fungi such as *H. marmoreus* (Zhang et al., 2015), *L. edodes* (Tang et al., 2016), and *Pleurotus eryngii* (Bakratsas et al., 2025), proteomic investigations addressing wavelength-specific effects remain limited. In this study, we analyzed the proteomic profiles of *H. erinaceus* exposed to different light qualities to elucidate its illumination-responsive behavior. The expression data revealed that *H. erinaceus* exhibits distinct responses to individual wavelengths, consistent with previous transcriptomic findings in related basidiomycetes. Notably, the canonical blue-light receptor proteins, White Collar-1 and White Collar-2 (Tisch and Schmoll, 2010), were not detected under blue-light treatment, suggesting that these receptors may be expressed at very low levels or are minimally active during the mycelial stage.

The analysis of protein expression under different light qualities revealed numerous DEPs in comparison with the control. Our observation that blue and RGB light induced the largest number of DEPs, while green light resulted in comparatively fewer changes, resonates with the well-established paradigm that fungi rely on specific photoreceptors to interpret spectral information. Among the DEPs, a core set was shared across all light treatments (blue, green, red, RGB), indicating that exposure to light initiates a baseline light-response module, regardless of wavelength (Zhang et al., 2022). These shared proteins likely govern basal adaptive processes such as redox balance, general stress responses, or energy redistribution, which may reflect a conserved mechanism of light adaptation across fungi (Fuller et al., 2013). Taken together, these results highlight that while each light wavelength imposes unique regulatory pressures on cellular pathways, the conserved core responses underscore the role of light as a fundamental environmental signal that orchestrates physiological stability and metabolic adjustment in *H. erinaceus* (Yu and Fischer, 2019; Yu et al., 2023). This dual pattern of wavelength-specific and universally conserved regulation further emphasizes the complexity of fungal photobiology and reinforces the importance of integrating multi-spectral cues in understanding fungal adaptation and metabolism (Shinohara et al., 2002; Fuller et al., 2015). Gene Ontology (GO) enrichment analysis further revealed that illumination strongly modulates metabolic and biosynthetic processes across all wavelengths. Blue light significantly up-regulated proteins involved in macromolecule biosynthesis, catalytic activity, and intracellular organelle function, indicating enhanced anabolic metabolism and intracellular reorganization under short-wavelength light. This agrees with previous findings in *Coprinopsis cinerea* and *Flammulina filiformis*, where blue light stimulates metabolic gene expression and promotes fruiting-body differentiation (Sakamoto et al., 2018; Wang et al., 2024). Similarly, red light-enriched pathways associated with nitrogen metabolism, biosynthesis, and cytoplasmic enzymatic activity, consistent with the well-documented role of fungal phytochromes in regulating energy metabolism and secondary metabolite pathways under red light (Wang et al., 2020). Green light generated more moderate enrichment patterns, suggesting modulation through indirect cross-talk rather than strong activation of dedicated photoreceptors, a concept proposed by fungal photobiology models (Corrochano, 2019). RGB light produced the broadest GO enrichment across metabolic, biosynthetic, and regulatory categories, indicating synergistic activation of multiple signaling pathways. This aligns with reports that fungi integrate multi-spectral cues to regulate development more robustly than under single wavelengths, producing emergent metabolic responses not observed under monochromatic light (Tisch and Schmoll, 2010). The analysis of the DEPs demonstrated that blue, green, red, and RGB light distinctly modulate specific KEGG pathways, with prominent effects on central metabolic processes, including genetic information processing and environmental information processing. Light-dependent regulation was also closely associated with pathways involved in microbial metabolism in diverse environments, starch and sucrose metabolism, pyruvate metabolism, and oxidative phosphorylation. These results underscore the role of light as a critical environmental signal in fungi, supporting previous studies that indicate fungal photoreceptors function primarily in regulating development, metabolic activity, and stress responses rather than energy acquisition (Corrochano and Garre, 2010; Kim et al., 2014; Corrochano, 2019).

### 4.1 Modulation of oxidative phosphorylation pathway by light in *H. erinaceus* mycelium

**Figure 10:**
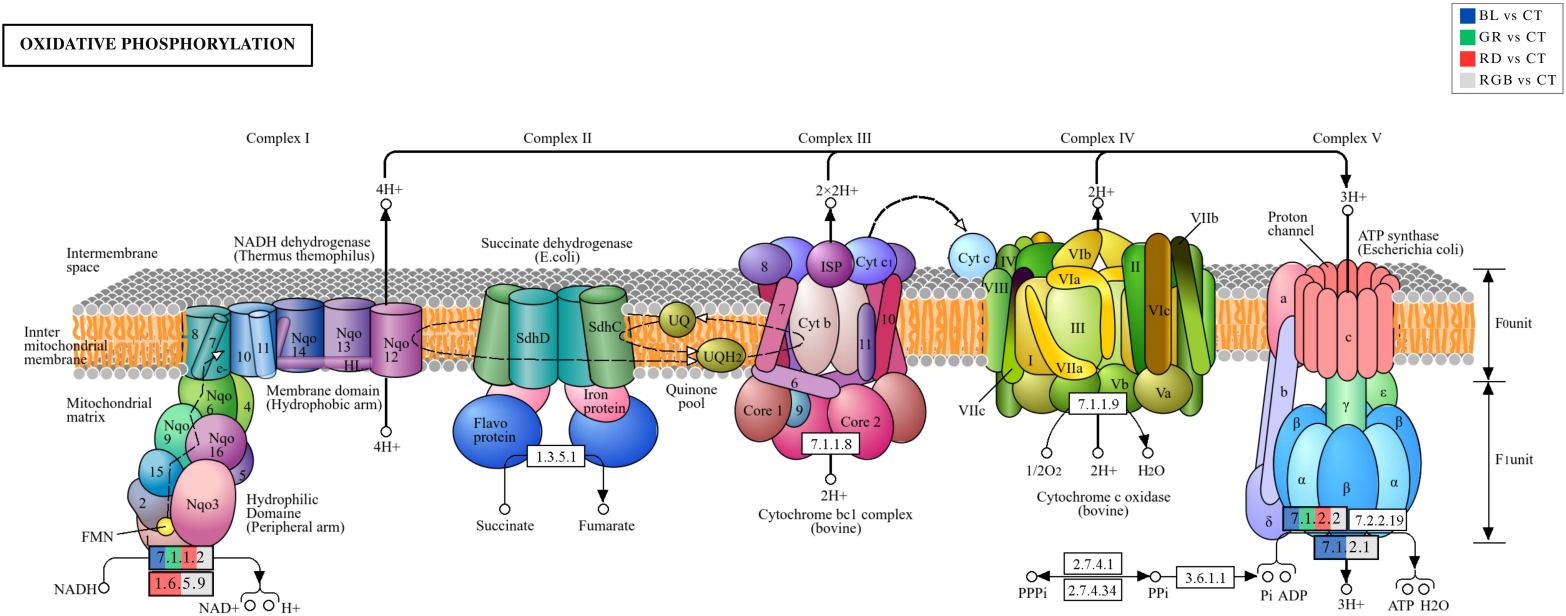
Differential expression analysis of proteins mapped to the oxidative phosphorylation pathway (ko00190) across all light treatments. Enzymes corresponding to DEPs identified in each comparison are highlighted using color-coded boxes (BL vs CT, GR vs CT, RD vs CT, and RGB vs CT). Differential expression was detected for enzymes including NADH: quinone reductase (EC:1.6.5.9; A0A4Y9ZY10), NADH dehydrogenase (EC:7.1.1.2; A0A4Y9XJD6, A0A4Y9ZBK7, A0A4Y9Y1T0, A0A4Z0A025), F-type H^+^-transporting ATPase subunit a (EC:7.1.2.1; A0A4Z0ABL1, A0A4Y9Y6H4), and F-type ATP synthase (EC:7.1.2.2; A0A4Y9ZAS5, A0A4Y9ZUI2).

In the present study, KEGG pathway enrichment analysis of DEPs revealed oxidative phosphorylation as a recurrently enriched pathway across all four light treatments (blue, green, red, and RGB)(**Figure 10**, adapted from Kanehisa et al. (2023)), indicating that mitochondrial energy metabolism is a major physiological target of light signaling in *H. erinaceus* mycelium (Kojima et al., 2015). Notably, DEPs were consistently mapped to EC:7.1.1.2 (NADH-quinone oxidoreductase subunit A) and EC:7.1.2.2 (H^+^-transporting ATPase), corresponding to NADH: ubiquinone oxidoreductase (Complex I) and F-type ATP synthase (Complex V), respectively, indicating that light exposure modulates mitochondrial energy metabolism at both electron entry and ATP synthesis levels (Lunova et al., 2020). Complex I serves as the primary entry point of electrons into the mitochondrial electron transport chain through NADH oxidation and proton translocation (Dröse et al., 2002; Wikström et al., 2023). The presence of DEPs at EC:7.1.1.2 across multiple light treatments suggests that light signaling influences cellular redox metabolism and respiratory activity in *H. erinaceus*. Blue light, in particular, is efficiently perceived by flavin-based photoreceptors such as the Cryptochromes, which are known to link light perception with transcriptional and metabolic regulation in fungi (Dröse et al., 2002; Liu et al., 2011; Tagua et al., 2015). Previous studies have demonstrated that blue light can alter NADH/NAD^+^ ratios, reactive oxygen species (ROS) balance, and mitochondrial respiration, supporting the observation that Complex I-associated proteins are responsive to illumination (Godley et al., 2005). Interestingly, enrichment at this enzymatic position was also detected under green and red light, as well as under RGB light, indicating that mitochondrial responses in *H. erinaceus* are not limited to canonical blue-light pathways. This suggests the involvement of additional photoreceptors, cross-talk between signaling pathways, or indirect metabolic feedback mechanisms that converge on mitochondrial function (Heintzen, 2012). Such convergence is consistent with reports that different light wavelengths can collectively influence fungal metabolism by modulating energy demand and redox homeostasis rather than activating entirely distinct pathways (Bakratsas et al., 2025). The identification of DEPs at EC: 7.1.2.2, corresponding to ATP synthase (Complex V), further indicates that light exposure affects not only electron transport but also the efficiency of ATP generation. ATP synthase converts the proton gradient generated by upstream complexes into ATP, making it a critical control point for cellular energy supply (Klusch et al., 2017). Changes in ATP synthase-associated protein abundance under light treatments suggest that *H. erinaceus* dynamically adjusts its energy production capacity in response to light-induced metabolic reprogramming. The simultaneous modulation of Complex I and ATP synthase supports the concept that oxidative phosphorylation is coordinately regulated to meet altered energetic and biosynthetic demands under illumination (Zhu et al., 2024a; Bakratsas et al., 2025). Light-induced growth responses, hyphal extension, and secondary metabolite biosynthesis in fungi are all energy-intensive processes, and modulation of ATP production may therefore represent an adaptive strategy to optimize resource allocation under different spectral conditions (Sarikaya-Bayram et al., 2014; Yu et al., 2023).

Additionally, blue and RGB light exposure in *H. erinaceus* mycelium, with DEPs specifically mapped to EC:7.1.2.1, corresponding to the H^+^-transporting ATPase subunit a of F-type ATP synthase (Complex V). This subunit forms the proton channel within the membrane-embedded F_0_ sector and is essential for coupling proton translocation to ATP synthesis, indicating that light exposure directly influences mitochondrial energy conversion efficiency (Kühlbrandt, 2019). Blue light is known to be perceived by flavin-based photoreceptors, which connect environmental light signals to transcriptional and metabolic regulation; modulation of ATP synthase components is therefore consistent with light-induced adjustments in cellular energy demand and mitochondrial function (Zhu et al., 2024a). The observation that RGB light elicited a similar response suggests that combined wavelengths may integrate multiple photoreceptor pathways or enhance signaling robustness, resulting in convergent regulation of ATP-producing machinery (Tisch and Schmoll, 2010). Rather than activating photosynthesis, these findings support the concept that light acts as an informational cue in fungi, fine-tuning oxidative phosphorylation to meet altered energetic requirements associated with growth, stress adaptation, or metabolic reprogramming (Corrochano, 2019; Zhu et al., 2024b). Collectively, the enrichment of EC 7.1.2.1 highlights ATP synthase as a key downstream target of fungal light signaling and underscores the central role of mitochondrial bioenergetics in mediating physiological responses of *H. erinaceus* to blue and RGB light. This includes exposure to red and RGB light in the *H. erinaceus* mycelium, with DEP mapped to EC:1.6.5.9, corresponding to NADH: quinone reductase (non-electrogenic). Unlike proton-pumping Complex I, this enzyme catalyzes the transfer of electrons from NADH to quinone without direct proton translocation, suggesting that red and RGB light modulate mitochondrial electron flow and redox balance rather than directly enhancing proton motive force (Gonçalves and Videira, 2015). Such modulation is consistent with the broader role of red light in fungi, which is often associated with regulation of cellular homeostasis, stress responses, and cofactor metabolism rather than strong stimulation of growth-related energy production. The enrichment of EC:1.6.5.9 under red light aligns with previous observations that red light can shift fungal metabolism toward redox regulation and NADH turnover, influencing cellular responses such as protein quality control, cell cycle regulation, and stress adaptation (Gil-Sánchez et al., 2022). The identification of this enzyme under RGB light further suggests a synergistic effect, whereby combined wavelengths integrate multiple photoreceptor-mediated signals and converge on mitochondrial redox processes. Rather than uniformly increasing ATP synthesis, RGB light appears to fine-tune respiratory electron flow, potentially balancing energy production with the prevention of excessive ROS generation (Marschall and Tudzynski, 2016; Verde-Yáñez et al., 2023). These findings support the concept that light acts as an informational cue in fungi, with different wavelengths eliciting distinct but overlapping metabolic responses.

### 4.2 Modulation of ribosome biogenesis in eukaryotes pathway by light in *H. erinaceus* mycelium

**Figure 11:**
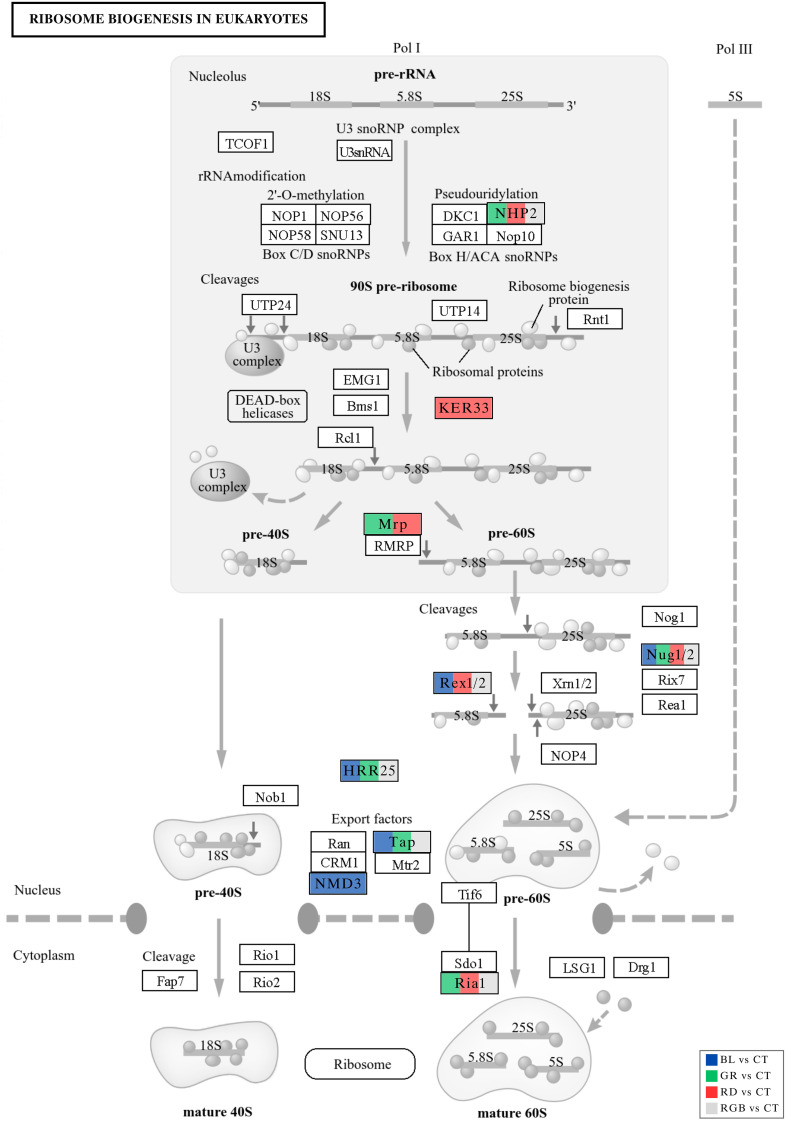
Differential expression analysis of proteins mapped to the ribosome biogenesis in eukaryotes pathway (ko03008) across all experimental comparisons. Enzymes corresponding to DEPs identified in each comparison are highlighted using color-coded boxes. Differentially expressed ribosome biogenesis related proteins include Nmd3 (A0A4Y9Y8W0), Rex2 (A0A4Z0A5M3), Tap (A0A4Y9YMR6), Nug2 (A0A4Y9XSA0), Nug1 (A0A4Y9ZHU4), Hrr25 (A0A4Z0AA76), Nhp2 (A0A4Z0ABH4), Mrp (A0A4Y9Y1J4), Ria1 (A0A4Y9ZVN4), and Ker33 (A0A4Y9ZNA1).

Ribosome biogenesis is a highly energy-demanding, multi-step process whose activity is coordinated with cellular metabolic status and environmental cues; regulation of assembly factors therefore provides a direct mechanism to match translational capacity with physiological demand (Warner, 1999; Dörner et al., 2023). The proteomic profiling of *H. erinaceus* under different light wavelengths revealed clear wavelength-specific modulation of key factors in ribosome assembly. Light quality exerted wavelength-specific regulatory effects on ribosome biogenesis in *H. erinaceus*, as shown by coordinated changes in DEPs including Nug1/2, NHP2, Ria1, HRR25, Tap, Rex1/2, Mrp, Nmd3, and Kre33 that map onto the KEDD ribosome biogenesis pathway (**Figure 11**, adapted from Kanehisa et al. (2023)). The nuclear GTP-binding proteins Nug1/2 were differentially expressed under blue, green, red, and RGB light, implicating modulation of late 60S subunit maturation. Nug2 (orthologous to Nug1/2 in yeast) acts as a checkpoint regulator on pre-60S particles, monitoring their maturation prior to export and preventing premature recruitment of the export adaptor Nmd3, which binds later and is essential for CRM1-mediated nuclear export (Matsuo et al., 2014). The differential expression of NHP2 under green, red, and RGB light highlights light-responsive control of early rRNA modification. NHP2 is a core component of the H/ACA snoRNP complex that catalyzes rRNA pseudouridylation, a critical step for proper folding and processing of pre-rRNAs. Disruption of snoRNP components is known to impair ribosome assembly and function (Henras et al., 1998; Ojha et al., 2020). Similarly, the expression of Ria1 under green, red, and RGB light functions in ribosome assembly steps associated with pre-60S remodeling (Bécam et al., 2001). Its modulation by light suggests wavelength-dependent effects on assembly efficiency and structural transitions necessary for export competence and subunit fidelity (Mitterer et al., 2023). The kinase HRR25, found as a DEP under blue, green, and RGB light, is a casein kinase I homolog implicated in multiple steps of ribosome assembly, including phosphorylation of assembly factors such as Ltv1, which regulates pre-40S maturation transitions (Strunk et al., 2011; Ghalei et al., 2015). HRR25-dependent phosphorylation promotes release of assembly intermediates and progression through maturation quality-control checkpoints (Mehlgarten et al., 2009; Ghalei et al., 2015). Nmd3, a Crm1-dependent 60S export adaptor, was strongly upregulated under blue light, suggesting enhanced nuclear export of pre-60S particles and elevated translational capacity, matching the KEGG enrichment showing that blue light stimulates ribosome biogenesis and mTOR/MAPK signaling, and consistent with the established role of Nmd3 in pre-60S export and cytoplasmic maturation (Sengupta et al., 2010). Proteins of the RNase P/MRP complexes, such as Mrp (under green and red light), are essential for processing precursor rRNAs and for proper assembly of the small subunit. Perturbations in RNase P/MRP function influence pre-40S formation and rRNA cleavage steps (Henras et al., 2015). The light-dependent expression of proteins like KER33, identified under red light, likely reflects modulation of accessory modification steps, such as N-acetylation of nascent peptides or ribosome-associated quality control factors that feed back into ribosome biogenesis (Ma et al., 2024). Although direct functional details of KER33 in ribosome assembly remain to be fully elucidated, such modifying enzymes are known to influence ribosomal protein stability and ribosome structural integrity.

Taken together, these findings demonstrate that light quality imposes differential regulatory pressure on ribosome biogenesis pathways in *H. erinaceus*. Blue light, which also drives the enrichment of energy metabolism and stress-responsive pathways in the broader proteome, appears to upregulate factors involved in late subunit maturation and export (Nug1/2, NMD3) and core kinases (HRR25) that accelerate quality-control transitions. The green light preferentially affects early rRNA modification (NHP2) and assembly factors (Ria1, HRR25), reflecting balanced growth conditions and a subtle modulation of translational capacity. Red and RGB lights influence a mixture of early processing, export, and RNA handling factors, suggesting an integration of environmental signaling with ribosome synthesis that may align with stress adaptation and translational reprogramming. This interpretation aligns with KEGG pathway annotation showing ribosome biogenesis as a hub of cellular regulation and confirms that ribosome assembly factors are responsive to environmental stimuli such as light spectra.

### 4.3 Modulation of the MAPK signaling pathway - yeast pathway by light in *H. erinaceus* mycelium

**Figure 12:**
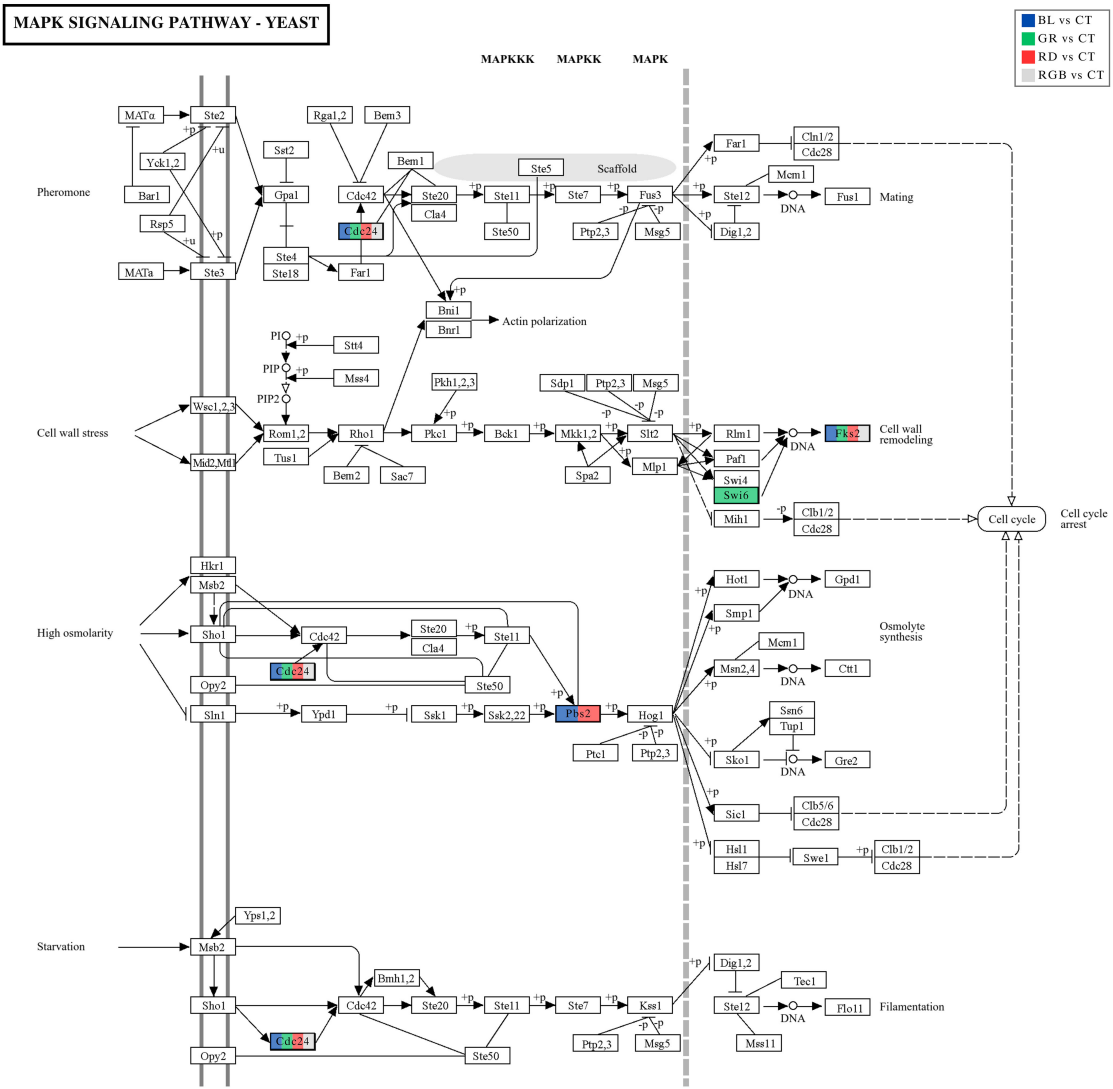
Differential expression analysis of proteins mapped to the MAPK signaling pathway-yeast (ko04011) across all experimental comparisons. Enzymes corresponding to DEPs identified in each comparison are indicated by color-coded boxes. Key MAPK pathway-associated proteins exhibiting differential expression include Cdc24 (A0A4Y9ZR78), Fks2 (A0A4Y9YSG8), Pbs2 (A0A4Y9ZT78), and Swi6 (A0A4Y9Y6T0).

The results of this study demonstrate that *H. erinaceus* mycelia undergo pronounced proteomic reprogramming in response to wavelength-specific light exposure, indicating that light quality functions as a key environmental regulator of intracellular signaling. Among the enriched pathways, mitogen-activated protein kinase (MAPK) signaling consistently emerged as a major environmental information processing pathway affected across multiple light treatments. MAPK cascades are fundamental eukaryotic signaling modules that enable cells to perceive, integrate, and respond to diverse environmental stimuli and stress conditions (Roux and Blenis, 2004; Zhu et al., 2022). In multicellular fungi, MAPK-related signaling has been implicated in the regulation of development, cell wall integrity, and environmental stress responses (Esquivel-Naranjo et al., 2016; Hu et al., 2019; Yang et al., 2021; Zhu et al., 2022). KEGG enrichment analysis of DEPs revealed that components of the MAPK signaling pathway were regulated under blue, green, red, and RGB light conditions compared with the control, indicating that fungal light perception is tightly coupled to intracellular signaling networks (**Figure 12**, adapted from Kanehisa et al. (2023)). However, it is important to note that KEGG pathway annotations are largely derived from yeast model systems, and signaling pathways cannot always be transferred directly across distantly related fungal lineages. Therefore, the MAPK components identified here should be interpreted as putative functional homologs or conserved signaling modules rather than exact pathway equivalents in *H. erinaceus*. Nonetheless, the recurrent enrichment of MAPK-associated proteins across multiple light conditions strongly suggests that core MAPK-like signaling architectures are involved in fungal light perception and intracellular signal transduction in this mushroom-forming species (Zhu et al., 2022). Within the MAPK pathway, two critical nodes, Cdc24 (cell division control protein 24) and Fks2 (1,3-β-glucan synthase 2), were identified as differentially expressed depending on light quality. Cdc24 functions as a guanine nucleotide exchange factor (GEF) for Cdc42, serving as a central upstream regulator that links external stimuli to cytoskeletal rearrangement, polarized growth, and activation of downstream MAPK cascades (Gulli et al., 2000; Harrell et al., 2024). The detection of Cdc24-related DEPs under all light treatments indicates a possible association between light exposure and pathways related to cell polarity and morphogenetic signaling in *H. erinaceus* (Corrochano, 2019). However, direct experimental validation of these mechanisms is required. This is consistent with the broader concept that fungi do not use light for photosynthesis but instead interpret light as informational input to regulate development and cellular organization through conserved signaling modules (Fuller et al., 2013). Notably, the MAPK branch associated with cell wall integrity (CWI) signaling showed evidence of modulation at the level of Fks2, a catalytic subunit of β-1,3-glucan synthase responsible for cell wall biosynthesis and remodeling (Levin, 2005). The Slt2/Mpk1 MAPK cascade transcriptionally and post-translationally regulates Fks2 and is typically induced under conditions of cell wall stress or altered growth demands (Levin, 2011). In this study, the presence of Fks2 among DEPs aligns with the strong enrichment of metabolic and growth-related pathways observed under these conditions. Enhanced energy metabolism and biosynthetic activity likely impose increased demands on cell wall synthesis, necessitating activation of CWI-associated MAPK signaling to maintain structural integrity during accelerated hyphal expansion.

In MAPK architecture, the MAPKKK-MAPKK-MAPK module converges at Pbs2, a mitogen-activated protein kinase kinase that relays signals to downstream MAPKs (e.g., Hog1) to regulate osmoadaptation, cell cycle arrest, and transcriptional defense programs (Maeda et al., 1995; Tatebayashi et al., 2020). By overlaying the proteomic DEPs obtained under blue and red light onto the MAPK pathway map, clear wavelength-specific regulatory patterns emerge. Blue light elicited the strongest metabolic and stress-related responses, marked by activation of oxidative phosphorylation, carbon metabolism, and MAPK signaling. The upregulation of Pbs2-associated proteins suggests that blue light functions as a potent stressor, likely increasing ROS and membrane stress and thereby intensifying HOG pathway activity (Molin et al., 2020). Corresponding enrichment of protein processing and ribosome biogenesis further indicates elevated cellular repair and biosynthetic demands under blue-light-induced physiological strain (Corrochano, 2019). In contrast, red light produced a more selective proteomic response, with enrichment of pathways related to cell cycle regulation, ubiquitin-mediated proteolysis, and redox-related metabolism. The involvement of Pbs2 under RD treatment appears linked not to broad metabolic activation but to more specific regulatory adjustments possibly associated with subtle shifts in membrane signaling or redox-sensitive osmotic balance (Tatebayashi et al., 2020). Under green light, the proteomic data indicate a moderate and targeted activation of MAPK signaling centered on the regulatory protein Swi6, which functions in cell cycle-associated transcriptional complexes (Koch et al., 1993; Horak et al., 2002). Unlike blue or red light, which elicited strong stress-responsive MAPK activation, green light primarily affected pathways related to oxidative phosphorylation, aminoacyl-tRNA biosynthesis, and nucleotide metabolism, suggesting maintenance of metabolic homeostasis rather than induction of stress adaptation (Bakratsas et al., 2025). The differential regulation of Swi6 therefore reflects green light’s role as a mild environmental cue that fine-tunes MAPK-dependent transcription and cell cycle progression, supporting steady biosynthesis and balanced physiological activity in *H. erinaceus* mycelium.

### 4.4 Light-dependent regulation of CAZymes in *H. erinaceus*

Carbohydrate-active enzymes (CAZymes) play a central role in fungal cell wall remodeling, nutrient acquisition, and environmental adaptation (Choi et al., 2015; Yang et al., 2021). In the present study, differential expression analysis of CAZymes under blue, green, red, and RGB light revealed pronounced wavelength-specific regulatory patterns across GHs, GTs, CEs, PLs, and AA enzymes in the mycelium of *H. erinaceus*. These results indicate that light quality functions as an important environmental cue shaping carbohydrate metabolism and cell wall–associated processes in this basidiomycete. Blue light exposure resulted in strong up-regulation of multiple GH family members, including GH47, GH16_1, GH13_22, and GH31_5, suggesting activation of pathways involved in polysaccharide hydrolysis and restructuring. GH16 and GH31 families are known to participate in β-glucan (Mouyna et al., 2016) and α-glucoside modification (Yuan et al., 2008), which are critical for fungal cell wall plasticity and hyphal growth. Similarly, GH47 enzymes are associated with N-glycan processing in the endoplasmic reticulum, linking blue light perception to protein maturation and secretion efficiency (Mast and Moremen, 2006). The preferential induction of GHs under blue light is consistent with previous studies demonstrating that blue light receptors, such as cryptochromes, regulate genes associated with cell wall biosynthesis, morphogenesis, and stress adaptation in fungi (Wei et al., 2025). Notably, AA family enzymes were consistently down-regulated under blue light, implying that oxidative degradation of polysaccharides is suppressed in favor of hydrolytic remodeling (Forsberg et al., 2011; Frandsen and Lo Leggio, 2016). This metabolic bias suggests that blue light promotes controlled cell wall turnover rather than aggressive oxidative depolymerization. Green light induces moderate and balanced CAZyme regulation compared with blue light. Green light elicited a milder transcriptional response, characterized by moderate up-regulation of selected GHs and CEs alongside down-regulation of some GHs and GTs (Im et al., 2024). CEs are involved in the deacetylation of hemicelluloses and other substituted polysaccharides, facilitating access of GHs to carbohydrate backbones (Liu et al., 2024). The concurrent but moderate activation of GHs and CEs under green light suggests maintenance-oriented carbohydrate metabolism rather than extensive structural remodeling. This balanced response aligns with emerging evidence that green light acts as a modulatory signal in fungi, fine-tuning metabolic homeostasis rather than triggering strong developmental or stress responses (Yu and Fischer, 2019). In *H. erinaceus*, green light may therefore support steady mycelial growth by sustaining basal carbohydrate turnover without inducing major oxidative or degradative pathways. In contrast to blue and green light, red light primarily enhanced the expression of AA family enzymes, including AA9, AA7, AA3_2, and AA3_3, while simultaneously down-regulating multiple GHs. AA enzymes, particularly lytic polysaccharide monooxygenases (LPMOs; AA9), catalyze oxidative cleavage of recalcitrant polysaccharides, generating new chain ends for further degradation (Frandsen and Lo Leggio, 2016). The preferential induction of AAs under red light suggests a metabolic shift toward oxidative carbohydrate processing. This observation is consistent with reports that red light influences mitochondrial activity and redox balance, potentially enhancing oxidative metabolism (Umino and Denda, 2023). In the context of *H. erinaceus*, red light may stimulate oxidative mechanisms to mobilize complex carbohydrate reserves or to remodel the cell wall under specific environmental conditions. The RGB light treatment produced the most diverse CAZyme expression profile, featuring up-regulation of certain GHs alongside broad down-regulation across GHs, GTs, CEs, and AAs. This heterogeneous pattern likely reflects simultaneous activation of multiple photoreceptors and signaling pathways, resulting in complex regulatory cross-talk (Yu and Fischer, 2019). Such mixed responses have been reported in fungi exposed to polychromatic light, where overlapping wavelength signals can generate competing transcriptional outputs (Corrochano, 2019). In *H. erinaceus*, RGB lighting appears to induce a non-linear regulatory state, potentially limiting excessive carbohydrate turnover while selectively enhancing specific hydrolytic functions. Collectively, these findings demonstrate that light wavelength is a critical regulator of CAZyme expression in *H. erinaceus*. Blue light favors GH-mediated hydrolytic remodeling, green light maintains balanced carbohydrate metabolism, red light promotes AA-driven oxidative processing, and RGB light induces complex regulatory interactions. These wavelength-dependent CAZyme profiles highlight the adaptive flexibility of *H. erinaceus* in modulating carbohydrate utilization and cell wall dynamics in response to environmental light cues.

Overall, this study provides comprehensive proteomic insights into wavelength-dependent light regulation in *H. erinaceus* mycelium, several limitations should be carefully considered when interpreting the findings. All experiments were conducted exclusively on PDA, a nutrient-rich, synthetic medium characterized by high readily available carbon content and relatively simple chemical composition. While PDA is suitable for controlled laboratory comparisons, it does not reflect the physicochemical complexity of natural or commercial cultivation substrates, which typically contain lignocellulosic materials, heterogeneous carbon sources, and structurally complex polysaccharides. Consequently, the light-responsive proteomic patterns observed here likely represent baseline regulatory responses under nutrient-sufficient conditions rather than the full adaptive capacity of the fungus in ecologically or industrially relevant environments. The exclusive use of PDA may particularly influence the interpretation of pathways related to CAZymes, oxidative phosphorylation, and MAPK signaling. On a simple glucose-rich medium, the demand for lignocellulose degradation is minimal; therefore, the regulation of glycoside hydrolases and auxiliary activity enzymes may differ substantially from expression profiles observed on wood-based or agricultural substrates. Similarly, mitochondrial bioenergetic adjustments detected under different light wavelengths may reflect modulation of metabolic flux under optimal growth conditions, rather than responses to substrate-induced stress, redox imbalance, or structural remodeling requirements encountered during solid-state cultivation. Thus, the extent to which light modulates energy metabolism and cell wall–associated pathways under complex substrate conditions remains to be validated. Future studies should therefore incorporate multi-factorial experimental designs integrating light quality with different cultivation substrates, including lignocellulosic materials, nitrogen-limited media, and liquid fermentation systems. Coupling proteomics with metabolomics and physiological measurements under these conditions would provide a more comprehensive systems-level understanding of fungal photoregulation. Validation at the fruiting stage and under semi-industrial cultivation settings will be particularly important to bridge mechanistic insights with practical applications. Such approaches will clarify whether the light-responsive pathways identified here represent conserved regulatory modules or context-dependent adaptations shaped by nutrient composition and developmental stage.

## 5 Conclusion

This study provides a comprehensive proteomic framework elucidating how wavelength-specific light cues modulate growth, metabolism, and regulatory networks in *H. erinaceus* mycelium. Using a label-free LC-MS/MS approach combined with functional enrichment analyses, we demonstrate that light acts as a potent informational signal that reprograms the fungal proteome in a wavelength-dependent manner, while maintaining a conserved core set of proteins associated with vegetative growth. Distinct illumination regimes induced characteristic proteomic signatures. Blue and RGB light exerted the strongest regulatory effects, driving extensive remodeling of metabolic pathways, ribosome biogenesis, and environmental information-processing systems, including MAPK signaling. These responses were accompanied by pronounced modulation of oxidative phosphorylation, indicating that mitochondrial energy metabolism represents a central downstream target of light perception. In contrast, red and green light elicited more selective proteomic adjustments, primarily affecting redox balance, stress-responsive pathways, and metabolic homeostasis, reflecting functional specialization of spectral signaling. Light-dependent regulation of ribosome biogenesis further highlights the tight coupling between environmental cues, translational capacity, and metabolic demand in *H. erinaceus*. Coordinated changes in ribosomal assembly factors suggest that light quality fine-tunes protein synthesis efficiency to support wavelength-specific physiological states. In parallel, differential expression of carbohydrate-active enzymes revealed that light governs polysaccharide metabolism and cell wall remodeling through distinct hydrolytic and oxidative strategies, underscoring the metabolic plasticity of this species. Collectively, our findings establish light quality as a key determinant of proteomic and metabolic organization in *H. erinaceus* mycelium. Rather than serving as an energy source, light functions as an environmental signal that integrates mitochondrial bioenergetics, translational control, signaling cascades, and carbohydrate metabolism. This study advances fundamental understanding of fungal photobiology in basidiomycetes and provides a molecular basis for optimizing light conditions in controlled cultivation systems aimed at improving biomass production and functional metabolite biosynthesis in medicinal mushrooms.

## Data Availability

The original contributions presented in the study are included in the article/**Supplementary Material**. Further inquiries can be directed to the corresponding author.
