# Supplementary material for: Comparative proteomic analysis reveals the effects of different light spectra on protein expression in Hericium erinaceus mycelium
Source: Front Fungal Biol. 2026 Mar 9;7:1791721. doi: 10.3389/ffunb.2026.1791721 (PMC13006603; doi:10.3389/ffunb.2026.1791721)
Supplement: Supplementary file 1 [file DataSheet1.pdf]

Supplementary Material

1 Supplementary Figures and Tables

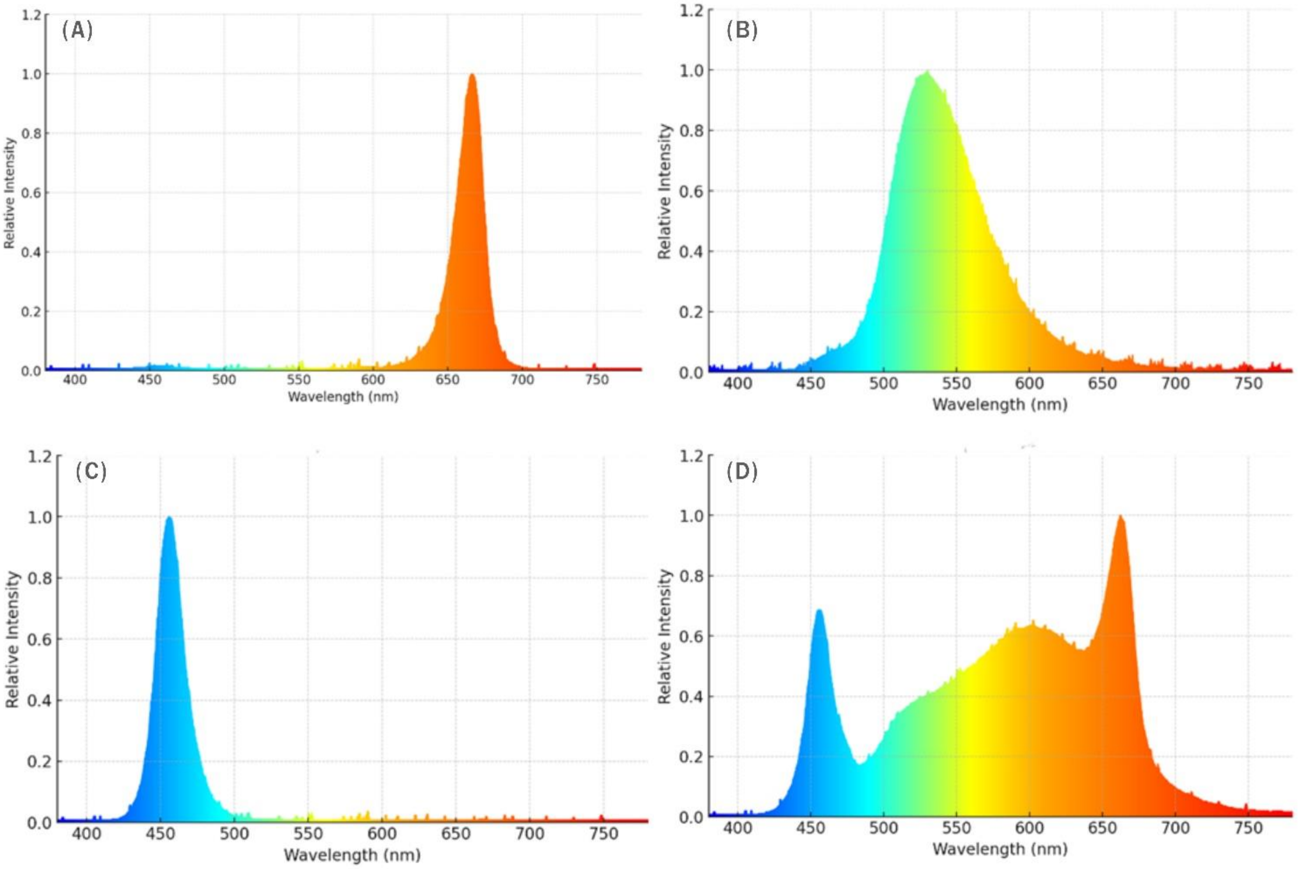

Supplementary Figure 1. Light spectrum graphs: A) Red light spectrum, B) Green light spectrum, C) Blue light spectrum, and D) RGB light spectrum. The spectral intensity of each light source was verified using a quantum light meter to ensure consistency
